# Transposon Mutagenesis of the Plant-Associated *Bacillus amyloliquefaciens* ssp. *plantarum* FZB42 Revealed That the *nfrA* and *RBAM17410* Genes Are Involved in Plant-Microbe-Interactions

**DOI:** 10.1371/journal.pone.0098267

**Published:** 2014-05-21

**Authors:** Anto Budiharjo, Soumitra Paul Chowdhury, Kristin Dietel, Barbara Beator, Olga Dolgova, Ben Fan, Wilfrid Bleiss, Jörg Ziegler, Michael Schmid, Anton Hartmann, Rainer Borriss

**Affiliations:** 1 Bakteriengenetik, Institut für Biologie, Humboldt Universität Berlin, Berlin, Germany; 2 Research Unit Microbe-Plant Interactions, Helmholtz Zentrum München, German Research Center for Environmental Health (GmbH), Neuherberg, Germany; 3 ABiTEP GmbH, Berlin, Germany; 4 Molekulare Parasitologie, Institut für Biologie, Humboldt Universität Berlin, Berlin, Germany; 5 Abteilung Molekulare Signalverarbeitung, Leibniz-Institut für Pflanzenbiochemie, Halle/Saale, Germany; Leibniz-Institute for Vegetable and Ornamental Plants, Germany

## Abstract

*Bacillus amyloliquefaciens ssp. plantarum* FZB42 represents the prototype of Gram-positive plant growth promoting and biocontrol bacteria. In this study, we applied transposon mutagenesis to generate a transposon library, which was screened for genes involved in multicellular behavior and biofilm formation on roots as a prerequisite of plant growth promoting activity. Transposon insertion sites were determined by rescue-cloning followed by DNA sequencing. As in *B. subtilis*, the global transcriptional regulator DegU was identified as an activator of genes necessary for swarming and biofilm formation, and the DegU-mutant of FZB42 was found impaired in efficient root colonization. Direct screening of 3,000 transposon insertion mutants for plant-growth-promotion revealed the gene products of *nfrA* and *RBAM_017140* to be essential for beneficial effects exerted by FZB42 on plants. We analyzed the performance of GFP-labeled wild-type and transposon mutants in the colonization of lettuce roots using confocal laser scanning microscopy. While the wild-type strain heavily colonized root surfaces, the *nfrA* mutant did not colonize lettuce roots, although it was not impaired in growth in laboratory cultures, biofilm formation and swarming motility on agar plates. The *RBAM17410* gene, occurring in only a few members of the *B. subtilis* species complex, was directly involved in plant growth promotion. None of the mutant strains were affected in producing the plant growth hormone auxin. We hypothesize that the *nfrA* gene product is essential for overcoming the stress caused by plant response towards bacterial root colonization.

## Introduction

Enhancement of plant growth by root colonizing bacteria is well-documented [1]. Plant-growth-promoting (PGP) bacilli have been commercialized as biofertilizers in recent years due to their ability to form thermostable and chemically resistant endospores, allowing preparation of durable products comparable to chemicals [2]. It is highly desirable to stepwise replace the use of environmental harmful agrochemicals (e.g. chemical fertilizers, and pesticides) by more environmental friendly biological agents. Although there is a tendency in this direction, the application of microorganisms is progressing slowly. This is probably due to the variable results obtained when microbial formulations are applied [3]. A way to overcome this difficulty is a better understanding of the basic principles in plant-PGPR interactions [4], [5]. Whereas a lot of progress has been made in this regard in gram-negative bacteria, e.g. *Pseudomonas fluorescens*, we still need to enhance our present knowledge about PGP bacilli [6].

FZB42 is the type strain for a group of plant-associated bacilli classified as *Bacillus amyloliquefaciens* subsp. *plantarum* [7], and is commercially available as bioinoculant. The sequencing of its genome revealed that FZB42 dedicates more than 8.5% of its total genetic capacity (3.92 Mbp) to non-ribosomal synthesis of bioactive secondary metabolites [6]. Secondary metabolites have strong antifungal and antibacterial activities and apparently enable the producing bacterium to survive in soil and thrive successfully to colonize the highly competitive rhizosphere habitat. Some of the mechanisms for these beneficial effects have been elucidated. For example, indole-3-acetic acid (IAA) and phytase production contributes to its plant growth promoting activity [8], [9]. In addition, volatiles emitted by *B. subtilis* and *B. amyloliquefaciens* enhance plant growth [10]. The non-ribosomal synthesized cyclic lipopeptides bacillomycin D and fengycin are able to inhibit growth of competitive phytopathogenic fungi such as *Fusarium oxysporum* in a synergistic way [11], whereas polyketide compounds and bacilysin act efficiently against bacteria such as the causative agent of fire blight disease *Erwinia amylovora* [12]. We could also show that FZB42 considerably reduced lettuce bottom rot caused by *Rhizoctonia solani* in a field naturally infested with this phytopathogen [13].

We have previously studied the colonization behavior of GFP-labeled derivatives of FZB42 on *Zea mays*, *Arabidopsis thaliana* and *Lemna minor*. Confocal laser scanning microscopy (CLSM) of the inoculated plant roots revealed that the bacterium colonizes plant species in different ways [14], [15], [16].

In this study, we performed transposon mutagenesis applying the mariner *Himar-1* transposon variant TnYLB-1 to identify genes involved in multicellular behavior and plant-growth promotion. We then used GFP labeled derivatives of those mutants to study their ability to colonize lettuce (*Lactuca sativa*) roots in a chemically defined axenic system. Besides identifying mutants affected in swarming and biofilm formation (*degU*, *yusV* and *pabA*), we provide evidence to suggest that two genes, namely *nfrA* and *RBAM_017141* are involved in plant-bacteria interactions.

## Materials and Methods

### Strains and growth conditions

Bacterial strains used in this study are listed in Table 1. *B. amyloliquefaciens* FZB42 was deposited as strain 10A6^T^ in the culture collection of the Bacillus Genetic Stock Center and as strain *B. amyloliquefaciens* DSM23117^T^ in the DSMZ culture collection [7]. All strains were grown in liquid Luria broth (LB) or solidified with 1.5% agar at 30°C. When necessary, appropriate antibiotics (ampicillin 100 µg/ml, kanamycin 5 µg/ml, erythromycin 1 µg/ml/lincomycin 25 µg/ml) were added to the medium. For biofilm formation screening, bacteria were grown in MSgg medium [17]. The duckweed clone *L. minor* ST was isolated by Pirson and Seidel [18], and was delivered from the culture collection of the Botanical Institute of Jena, Germany. *L. minor* ST was propagated axenically in filter sterilized Steinberg medium as described previously [9].

**Table 1:** Bacterial strains, plasmids and primers used in this study.

Strain	genotype	description	source/reference
FZB42	Wild-type	Producer of lipopeptides and polyketides	BGSC 10A6
FB01mut	FZB42 *amyE::gfp^+^-emR*	Labeled by green fluorescent protein	14
AB101	FZB42 *pabA*::TnYLB-1	Impaired in biofilm formation	TnYLB-1→ FZB42
AB102	FZB42 *yusV*::TnYLB-1	Impaired in biofilm formation	TnYLB-1 → FZB42
AB103	FZB42 *degU*::TnYLB-1	Impaired in swarming and biofilm formation	TnYLB-1 → FZB42
AB106	*FZB42 nfrA*::TnYLB-1	Impaired in plant-growth-promotion	TnYLB-1 → FZB42
AB107	FZB42 *RBAM_017410*::TnYLB-1	Impaired in plant-growth-promotion	TnYLB-1 → FZB42
AB108	FZB42 *abrB*::TnYLB-1	Impaired in plant-growth-promotion	TnYLB-1 → FZB42
BB3	FZB42 *amyE:: gfp^+^-em^R^ degU::kmR*	Labeled by green fluorescent protein	AB103→ FB01mut
BB4	FZB42 *amyE:: gfp^+^-em^R^ pabA::kmR*	Labeled by green fluorescent protein	AB101→ FB01mut
BB5	FZB42 *amyE:: gfp^+^-em^R^ yusV::kmR*	Labeled by green fluorescent protein	AB102→ FB01mut
AB2	FZB42 *amyE:: gfp^+^-emR nfrA::kmR*	Labeled by green fluorescent protein	AB106→ FB01mut
AB4	FZB42 *amyE:: gfp^+^-emR RBAM_017410::kmR*	Labeled by green fluorescent protein	AB107→ FB01mut
AB9	FZB42 *amyE:: gfp^+^-emR abrB::kmR*	Labeled by green fluorescent protein	AB108→ FB01mut
**Plasmids**			
pGEM-T	ApR	Promega cloning vector	Promega
pMarA	promoter σA, kmR ApR EmR	pUC19 carrying TnYLB-1 transposon, mariner-Himar1 transposase	19
pMarB	promoter σB, kmR ApR EmR	pUC19 carrying TnYLB-1 transposon mariner-Himar1 transposase	19
pMarC	kmR ApR EmR	pUC19 carrying TnYLB-1 transposon mariner-Himar1 transposase	19
pVBF	*amyE lacZ′* ApR	pUC18 carrying *amyE* fragment	14
pAB1	FZB42*pabA*	pVBF carrying *pabA* fragment	This study
pAB2	FZB42*yusV*	pVBF carrying *yusV* fragment	This study
pAB3	FZB42*degU*	pVBF carrying *degU* fragment	This study
pAB6	FZB42*nfrA*	pVBF carrying *nfrA* fragment	This study
pAB7	FZB42*RBAM_017410*	pVBF carrying *RBAM_017410* fragment	This study
pAB8	FZB42*abrB*	pVBF carrying *abrB* fragment	This study
**Primers**	**Restriction site/use**	**Sequence (5′to 3′end)**	
oIPCR1	IPCR experiments	GCTTGTAAATTCTATCATAATTG	19
oIPCR2	IPCR experiments	AGGGAATCATTTGAAGGTTGG	19
oIPCR3	IPCR DNA sequencing	GCATTTAATACTAGCGACGCC	19
yusV-dw	Eco911/*vusV* amplification	CTCCCATTGGAATTTGGACAGCCGCTATGAC	This study
yusV-up	SacII/*vusV* amplification	AGCCCGCGGTCCGTGTATTTCTCAAGCAGG	This study
nfrA-dw	Eco881/*nfrA* amplification	AATCCCGAGATCGAATCGTTTCATTCCTCG	This study
nfrA-up	ClaI/*nfrA* amplification	TTATAGCTATTCACACCTTCCAGAACATCG	This study
1741-up	SacII/*RBAM_017410* amplification	AACCCGCGGATTGCATTGAACGGCGGTCT	This study
1741-dw	Eco911/*RBAM_017410* amplification	GCACCATTGGATCCCTTTGGTATCCCTCAG	This study
degU-dw	ClaI/*degU* amplification	AATATCGATTCACCGAAAACCACTTGGAG	This study
degU-up	Eco881/*degU* amplification	ATACCCGAGTAGGATAAGGAGGCGTAGCG	This study
pab-dw	Eco911/*pabA* amplification	TTTGGTTACCTGAATAGAGACATACACACGGC	This study

### Transposon mutagenesis in *B. amyloliquefaciens*

The *mariner* based transposon TnYLB-1 plasmid was used to generate a transposon library according to Haldenwang (Le Breton *et al.* 2006). Plasmids pMarA and pMarB differ in the promoters that drive the expression of the *Himar1* transposase gene. pMarA has *Himar1* under the transcriptional control of housekeeping σ factor σ^A^ of *B. subtilis*, while pMarB B uses the general stress response σ factor σ^B^ for transposase expression. Plasmid pMarC has no transposase gene as well as its promoter and is used as a control [19]. In brief, plasmids pMarA, pMarB and pMarC were transformed into *B. amyloliquefaciens* FZB42. Competent cells of *B. amyloliquefaciens* were obtained by modifying the two-step protocol of Kunst and Rapoport [20] according to Idris et al. [9]. Transformants were screened for plasmid-associated properties, i.e. Kan^r^ and Erm^r^ at permissive temperature for plasmid replication (30°C) and Kan^r^ and Erm^s^ at the restrictive temperature (48°C). To verify that these transformants contained the original intact plasmid the plasmid was extracted from the transformants and transformed into *E. coli* DH5α. Plasmid DNA was extracted from *E. coli* DH5α and subjected to restriction endonuclease analysis with *EcoRI*. The restriction was then analyzed through agarose gel electrophoresis to verify that the transformants contained the correct plasmid. (Fig. S1). For inducing transposition, isolated clones were grown overnight in liquid LB medium at 37°C, and then portions of each culture were plated on either LB, LB and Kan (5 µg/ml), LB and Erm (1 µg/ml) and incubated at non-permissive temperature for plasmid replication (48°C) to select for transposants as described previously [19].

For mapping of transposon insertion sites, five micrograms of genomic DNA isolated from the transposant, was digested with *Taq* I. The reaction mixture was circularised in a ligation reaction using ‘Rapid Ligation’ kit (Fermentas, Germany) at a DNA concentration of 5 ng/µl. Inverse PCR was performed with 100 ng of ligated DNA using primers oIPCR1 and oIPCR2, which face outward from the transposon sequence. IPCR products were purified using PCR purification kit (Amersham, UK) and sequenced using the primer oIPCR3 (Table 1).

### Southern hybridization

For each Southern hybridization, an appropriate probe was labeled with Digoxigenin-11-dUTP (DIG-dUTP), according to the Ready-to-Go kit from Roche. Prior to labeling, the desired DNA region was amplified by PCR and purified. 100 ng of the PCR fragment were denaturated by heating at 100°C for 10 minutes and then mixed with 5 µl dCTP (10 mM), 2.5 µl DIG-dUTP (1 mM) to a final volume of 50 µl. The mixture was incubated at 37°C for 1.5 hours and was stored at -20°C until use. One to two µg of the chromosomal DNA were digested overnight with a suitable restriction endonuclease. Samples were initially separated on a 0,8% agarose gel in 1× TAE buffer at 70 Volt. The gel was washed twice for 20 minutes, initially with a denaturation buffer (1.5 M NaCl, 0.5 M NaOH) and subsequently with neutralization buffer (1.5 M NaCl, 1 M Tris/HCl pH 8.0). Transfer to a nylon membrane was performed using the Biorad vacuum blotter (model 785). The DNA was fixed permanently on the membrane by cross-linking using UV radiation. The membrane was initially incubated for 1 hour at 65°C with 40 ml hybridization buffer and was hybridized overnight at 55°C with 5–10 ml hybridization buffer containing 5–25 ng/ml of denaturated DIG-labelled probe. The membrane was washed twice for 15 minutes, first with 2× SSC/0,1% SDS at room temperature and then with 0,5× SSC/0,1% SDS at 55°C. Detection was achieved by a colorimetric approach. The membrane was first equilibrated with P1-DIG buffer and was then incubated for 30 minutes with P1-DIG buffer containing 3,75 units of the antibody Anti-Digoxigenin-Alkaline-Phosphatase. Unbound antibody was removed after a 15 minute washing step. Addition of 10 ml Ap buffer containing 2,25 mg nitroblue tetrazolium salt (NBT) and 1,75 mg 5-bromo-4-chloro-3 – indolyl phosphate (BCIP) to the membrane followed by an incubation period in the dark allowed visualization of the hybridized DNA with our labeled probe.

### Screening for swarming and biofilm formation

Swarming was determined on 0.9% LB agar plates overnight at 30°C. Putative mutants, impaired in swarming, were identified as small colonies and then transferred onto mini Petri dishes (30 mm diameter) containing 5 ml swarming agar (LB solidified with 0.7% agar). While wild-type cells completely spread over the whole surface of the Petri dish, mutants impaired in their swarming capability did not. Further confirmation of reduced swarming motility was accomplished by incubating the candidate clones for 24 hrs at 37°C onto swarming agar [21].

Mutants impaired in biofilm formation were identified by inoculating of the transposants into 96-well microtiter plates filled with 140 µl LB medium/well. The microtiter plates were shaken at low speed (160 rpm) at 37°C for 16 hrs. Then, 5 µl of each culture was transferred into 1 ml MSgg medium within a 48-well microtiter plate. The microtiter plates were incubated without shaking at 30°C for 60 hrs and development of biofilm was analysed by visual inspection [17].

### Screening for plant-growth-promotion

We used the *Lemna* biotest system for plant-growth-promotion assays [9]. Four plants with two or three budding-pouches (fronds) were grown in 200 ml Steinberg medium. The flasks were kept at 22°C with continuous light until a sufficient number of homogenous *Lemna* plantlets were obtained. *Lemna* plantlets with two fronds were transferred aseptically into 48-well-microtiter plates filled with 1.25 ml/well. 1 µl of growing wild-type or mutant culture (OD_600_ 1.0) was added directly. The growth medium was replaced every week. The microtiter plates were kept at 22°C and 24 h light for 10 days. Subsequently, the plants were harvested and the dry weight was determined from four repetitions.

An additional test system based on *Arabidopsis thaliana* was used for corroborating the results obtained with *L. minor*. The seeds were surface sterilized with 10% sodium hypochlorite solution for three minutes. To remove the bleach, sterile distilled water was added, and changed four times. The surface sterilized seeds were pre-germinated on Petri dishes containing half-strength Murashige-Skoog medium (50%) with 0.6% agar and incubated at 22°C under long day light conditions (16 h light/8 h dark) for seven days. Then, the roots of seven day-old *Arabidopsis* seedlings were dipped into a diluted bacterial spore suspension (1×10^5^ CFU/ml) for five min. and four seedlings were transferred into a square Petri dish containing half-strength MS-medium solidified with 1% agar. The square Petri dishes were incubated in a growth chamber at 22°C at a daily photoperiod of 14 hrs. Fresh weight of the plants was measured 21 days after transplanting.

### Strain construction

In order to complement the genes disrupted by TnYLB-1 insertion through the respective wild-type genes, appropriate gene cassettes were constructed using the primers listed in Table 1. To complement the disrupted *degU* mutant gene, the coding region plus 178 bp of the upstream sequence and 183 bp of the downstream sequence was amplified using primers degU-dw and degU-up. The amplified 889 bp fragment was introduced into ClaI/*Eco881* digested plasmid pVBF, and then transformed into *E.coli* DH5α cells. To confirm presence of the *degU* gene, PCR using the degU-dw-ClaI and degU-up Eco881 primers was performed. The resulting plasmid pVBFdegU was transformed into the mutant with the disrupted *degU* gene, AB103. The transformants were grown in LB + Kan (5 mg/L) + Erm (1 mg/L) and 1% starch. The transformants without starch hydrolysis were analyzed for containing the wild-type *degU* gene.

The intact *yusV* gene plus 77 bp upstream sequence and 170 bp downstream sequence was amplified using primers yusV-up-SacII and yusV-dw-Eco911. The 1072 bp fragment was cloned into pVBF digested with *SacII* and Eco911. The ligated plasmid DNA was transformed into competent DH5α cells. The plasmid containing the correct *yusV* insertion was transformed into the *yusV* insertion mutant AB102. Clones unable to hydrolyse starch and able to grow onto Kan and Erm containing LB agar were checked for the presence of the wild-type *yusV* gene (Fig. S2).

To complement the *pabA* gene that has been disrupted by the insertion of TnYLB-1, a plasmid harboring the intact *pabA* gene from FZB42 was constructed using primers pab-dw Eco911 and pab-up-SacII. The amplified fragment was ligated into the pVBF vector digested with *Eco911* and *SacII*. The resulting recombinant integration plasmid was transformed into AB101, bearing the *pabA* disruption, to complement the wild-type *pabA* genotype.

Complementation of the *nfrA* gene was done by amplifying the *nfrA* coding region plus 241 bp upstream and 278 bp downstream sequences using nfrA-dw-Eco881 and nfr-up-ClaI primers. The resulting 1269 bp product was cloned into the pVBF vector after digesting with *ClaI* and *Eco881*. The recombinant plasmid bearing the correct insert was used to complement the *nfrA* disruption in AB106.

To complement the *RBAM_17140* mutant, the *RBAM_017410* gene (186 bp) plus 261 bp upstream region and 123 downstream region was amplified using the 410-up-SacII and 410-dw-Eco911 primers. The amplified fragment (586 bp) was ligated into the *Sac*II/*Eco*911 digested plasmid pVBF. The ligation mixture was transformed into DH5α cells. We introduced the plasmid with the correct insert into competent AB107 cells to complement the disrupted *RBAM_17410* gene.

In order to exclude that the mutant phenotype was due to a combined effect of multiple TnYLB-1 insertions, FZB42 wild-type strain was transformed with chromosomal DNA isolated from AB101, AB102, AB103, AB106, and AB107. The retransformed strains were found to have the same phenotype as the original mutant strains.

To construct the gfp-labeled mutant strains, chromosomal DNA isolated from AB101, AB102, AB103, AB106, and AB107 was prepared to transform competent cells of strain FB01mut, a *B. amyloliquefaciens* FZB42 derivative, expressing green fluorescent protein, GFP [14]. Chromosomal DNA from GFP expressing transformants was prepared and used for PCR with the appropriate primer pairs for detecting the respective genes. Kan^R^ clones with the correct size of the amplified fragments indicating TnYLB-1 insertion were selected (Table 1). The Gfp expressing strains showed the same phenotype as the original strains including their growth kinetics.

### Rhizosphere colonization assay

*Lactuca sativa* cv. Tizian seeds were surface sterilized by washing with 1% Tween 80 for 2 min and 13% sodium hypochloride for 10 min with washing with sterile water (3 times) in between. The sterilized seeds were placed on nutrient agar plates and allowed to germinate in the dark for two days and checked for bacterial or fungal contamination. Pre-germinated lettuce seeds were soaked in a spore suspension of GFP-labeled *B. amyloliquefaciens* FZB42 mutants (Table 1) at a concentration adjusted to 10^7^–10^8^ cfu/mL with 0.3% NaCl for one hr at room temperature. Inoculated seedlings were transferred to sterile plastic trays (Phytatray, Sigma) with sterilized quartz sand and 20 ml of Hoagland's solution (Sigma). Plants without bacterial inoculation were used as control. The trays were placed in growth chamber (Vötsch BioLine VB 1514) at 18°/20°C with 12-h light-dark intervals.

To determine the CFU/g root fresh weight of the inoculants, three inoculated seedlings for each treatment were analyzed after one week of growth. The root systems were weighed aseptically to calculate the root fresh weight and then were vortexed for 30 seconds in an Eppendorf tube containing one ml sterile saline. Serial dilutions were spread plated on LB agar plates and were incubated at 30°C overnight. The number of individual colonies was counted and the collected data were analyzed.

### Confocal Laser Scanning Microscopy (CLSM)

CLSM was performed with LSM 510 microscope (Carl Zeiss, Jena) using an excitation wavelength 488 nm (Argon laser) and collecting the emission band of 500–550 nm for GFP flourescence. Emissions obtained using wavelengths of 543 nm and 633 nm (Helium neon lasers) were used as control to visualize root structure. Images were acquired and reconstructed using Zeiss LSM Image browser.

### Electron Microscopy

For scanning electron microscopy (SEM) the *Arabidopsis* roots inoculated with FZB42 and *nfrA* mutant cells were processed as described previousy [14]. Dehydration, through a gradued series of ethanol solutions and finally 100% acetone, was followed by critical point drying with liquid carbon dioxide using the CPD030 (BAL-TEC, Germany). Specimen were then mounted on stubs for SEM, sputtered with gold (Sputter Coater SCD, 005, BAL-TEC, Germany) and examined with a LEO 1430 scanning electron microscope.

### IAA measurements

Cells were cultivated as described previously [9]. After centrifugation of the bacterial suspension, 250 µl of the supernatant was supplemented with 200 µl MeOH containing 5 ng [^13^C_6_]-IAA as internal standard. The final supernatant was diluted with 1 ml aqueous formic acid solution (2% v/v) and subjected to solid phase extraction (SPE). The SPE 96-well plate was prepared by distributing dry HR-XC-resin (Macherey-Nagel, Düren, Germany) into the wells of a 96-well filtration plate (50 mg per well). The resin was conditioned by 1 ml of methanol followed by 1 ml of water. In this and all subsequent steps, the liquid was passed through the resin by centrifugation at 250 g for 5 min using a JS5.3 swing-out rotor in an Avanti J-26XP centrifuge (Beckman Coulter, Fullerton, CA, USA). After loading the samples, the resin was washed with 1 ml of water, and the analytes were eluted with 1 ml of methanol into a 96 deep well plate (Roth, Karlsruhe, Germany). The eluates were loaded onto a 96-well plate filled with DEAE-Sephadex A-25 (acetate form, 50 mg/well, GE-Healthcare BioSciences, Uppsala, Sweden). After washing with 1 ml of methanol, the analytes were eluted with 3 M acetic acid in methanol. The eluates were transferred to 2 ml Eppendorf vials and the solvent was evaporated under vacuum in a Savant SC210A Speed Vac Concentrator at 45°C (ThermoFisher Scientific, Waltham, MA, USA). The dry residue was dissolved in 40 µl 20% (v/v) methanol. After dilution with 40 µl of water and centrifugation at 10,000 g for 10 min, the samples were transferred to autosampler vials for LC-MS/MS analysis.

Separations were performed on a Nucleoshell RP18 column (50×3 mm, particle size 2.7 µm; (Macherey-Nagel, Düren, Germany) at 30°C using an Agilent 1290 Infinity HPLC system. Eluents A and B were water and acetonitrile, respectively, each containing 0.2% (v/v) acetic acid. After an initial hold at 10% B for 0.5 min, the percentage of B was increased to 40% over 4.5 min, further increased to 98% in 1 min followed by an isocratic period of 1.5 min at 98%B. The starting conditions were restored within the next 0.5 min, and the column was allowed to re-equilibrate for 1 min at 10% B. The flow rate was set to 0.5 ml/min. IAA was detected on-line by ESI-MS/MS using an API 3200 triple-quadrupole LC-MS/MS system equipped with an ESI Turbo Ion Spray interface, operated in the negative ion mode (AB Sciex, Darmstadt, Germany). The ion source parameters were set as follows: curtain gas: 50 psi, ion spray voltage: −3500 V, ion source temperature: 650°C, nebulizing and drying gas: 70 psi and 50 psi, respectively. Triple quadrupole scans were acquired in the multiple reaction monitoring mode (MRM) with Q1 and Q3 set at “unit” resolution. Scheduled MRM was performed with a window of 60 s and a target scan time of 0.1 s. Peak areas were calculated automatically using the IntelliQuant algorithm of the Analyst 1.6.2 software (AB Sciex, Darmstadt, Germany) and manually adjusted if necessary. All subsequent calculations were performed with Excel (Microsoft Office Professional Plus 2010). IAA was quantified using the internal standard.

## Results

### Transposon mutagenesis in *B. amyloliquefaciens* FZB42

To mutagenize FZB42, we used plasmids bearing a thermo-sensitive (*ts*) origin of replication and the erythromycin resistance gene, which have been developed for *B. subtilis* based on the mariner *Himar-1* transposon variant TnYLB-1 [19]. The plasmids were separately transformed into competent FZB42 cells [20], and plated on Kan (5 µg/ml) containing agar, and incubated at 30°C. While the plasmids pMarA and pMarC were successfully transformed into FZB42, for unknown reasons, no Kan^R^ pMarB transformants were obtained. We then used pMarA transformants for preparing the transposon library, as pMarA and pMarB contained the same *Himar-1* mariner transposase gene but differed in their promoters.

pMarA transformants were verified as described in the Materials and Methods section for the presence of an intact plasmid before being used for transposon mutagenesis. After inducing transposition, Kan^r^ colonies, which indicated transposition events, appeared at a very high frequency of nearly 8×10^−2^. Less than 2% of the temperature-resistant Kan^R^ clones grew at 48°C on Erm containing agar. By contrast to pMarA transformants, there were no antibiotic-resistant clones detectable when *B. amyloliquefaciens* FZB42 pMarC transformants were streaked out on either Kan or Erm containing agar at 48°C. This suggests that 98% of the Emr^r^ clones obtained after transformation with pMarA were a consequence of true transposition events, and less likely a result of mutations through which the temperature-sensitive plasmid replication has become temperature-resistant. Representative data that are the average of two independent experiments are shown in Table 2.

**Table 2:** TnYLB-1 transposition in FZB42. CFU resulting from plating of two separate overnight (O/N) cultures of *B. amyloliquefaciens* strains carrying either pMarA, or -C at 50°C on LB agar with or without the indicated antibiotics.

Delivery plasmid	Viable cell number (CFU/mL)			Transposition frequency	Erm^R^/Kan^R^
	LB 48°C	LB Kan^R^ 48°C	LB Erm^R^ 48°C		
pMarA	3.4×10^8^	2.6×10^7^	3.6×10^5^	7.6×10^−2^	1.4%
pMarC	2.5×10^8^	0	0	-	-

Transposition frequency was calculated as Kan^r^ colonies/LB colonies. Erm^r^/Kan^r^ represents the percentage of the O/N cultures that displayed the plasmid-encoded antibiotic resistance (Erm^r^) versus the transposon-encoded resistance (Kan^r^). The data presented in Table 2 are the average of two representative, independent experiments.

We performed Southern blot analysis to verify transposon integration and to test whether the insertions are likely to be random. In this analysis, chromosomal DNA from *B. amyloliquefaciens* FZB42 and 10 clones that had been selected after plating FZB42/pMarA at 48°C were isolated and digested with *Eco*RI. *Eco*RI cut pMarA twice, releasing a 4.5-kb fragment containing the transposon, but did not cut within the transposon itself. If the transposon had inserted randomly into the chromosome in these 10 clones, a transposon-specific probe would be expected to hybridize to a single, uniquely sized DNA fragment (Le Breton et al. 2006). Digoxigenin-labeled DNA specific for the transposon was prepared by digesting the TnYLB-1 region with *Ps*tI. Hybridization of this probe with the *Eco*RI-digested DNAs from the transformants resulted in variable band patterns suggesting that transposon insertions were random (Fig. S3).

A number of 787 temperature-resistant Kan^r^ clones were spotted onto a glucose minimal medium to screen for auxotrophic mutations. Seven out of the 787 spotted clones failed to grow on the minimal medium. Further investigations were then executed in two out of these seven auxotrophic clones and they were found to harbor TnYLB-1 insertions within the *hisJ* and the *pabA* genes (Table 3). The addition of histidin and para-aminobenzoic acid in the minimal medium restored the growth of the *hisJ* and *pabA* auxotrophs, respectively.

**Table 3:** Gene sequences downstream from the TnYLB-1 insertion site (bold face) in one auxotrophic clone (*hisJ*), three mutants impaired in multicellular behavior (AB101, AB102, AB103), and three mutants selected for reduced plant-growth-promotion (AB106, AB107, AB108).

Strain	Gene	Genome location	Insertion site	Gene disruption at
		nucleotide	nucleotide	nucleotide/amino acid
Aux 1	*hisJ (RBAM_026550)*	2,784,334…2,785,146	2,784,653	255/S85
2,784,653	**TA**CGTGTTCAGAAACGCCGCGGTTTCCTCTTCATACCCTGTGATAAAATCGGTTTCAAGTCCAGTCAGAATGCTGATTTGTCCATCGTATTCCTTT… 2784558			
AB101	*pabA (RBAM_000860)*	84,919… 85,506	84,929	10/M4
84,927	**TA**AAATCATCTCTGCTCACCTCGTCTAATTTTTTGTCTCTTCTTCGCTCAGCTCTAACGCCTTTTTCATTGCAAAAGCTTTTTTGAACGATTCCCTTT… 84,834			
AB102	*yusV (RBAM_030060)*	3,122,023…3,121,199	3,121,518	506/T168
3,121,518	**TA**GGTTGTCGGCTCGTCAAGCAGAATGATATCCGTATCCTGAGCGAGTGTCATCGCAATCCATGCGCGCTGGCGCTGTCCGCCTGACAAGGAAT… 3,121,611			
AB103	*degU (RBAM_032640)*	3,380,502…3,379,813	3380014	489/L163
3,380,014	**TA**ATGGTCTCCGGATTTCCGGGTAAACCTCATGCTGAGGATGAGCAGAAACTCCGCTTGTCGCAAGACGGCGGAATTCGTTGACAAGATTGTGG… 3,380,107			
AB106	*nfrA (RBAM_035360)*	3,641,993… 3,641,244	3,641,834	161/K54
3,641,834	**TA**TCAGTTACGCCGATGATGGAGTAAGCCTGCACGTAACTGGACGTTGACGCCGCCTGGGCGCTTTTCACTAATGTATCTATCTCTTCTGCTGTC… 3,641,928			
AB107	*RBAM_017410*	1,809,412… 1,809,597	1,809,543	132/D44
1,809,543	**AT**CCATTCTCCCCACAGGCATCTTCTTCACACTCGGAAGTGTAAGGTAAGCTGTTATCTCATCCCCTGATTTTAATGTTTTTCCGCAGATAGAGCAT… 1,809,447			
AB108	Upstream region *abrB*	45,896…46,384	46,308	-
46,308	**AT**ATTTGTCCTCTATCTATGATACCATCTTAGCCTTTAAGATCAAGGATATATACGTACTGCGCGTAAAAGAGTAAATAAAATTAAAAATTTTTATTG… 46,210			

The T residues either 5 or 6 bps distant from the insertion site are underlined.

### Isolation of swarming and biofilm mutants

The data presented in the previous section strongly suggests that the *Himar1-*based transposon system developed by the Haldenwang group for *B. subtilis* [14] is effective in FZB42. Consequently, this system might be useful for identifying genes involved in plant-growth-promotion (PGP) and root colonization. We have shown recently that FZB42 spores applied to plant roots start to germinate, and that the arising vegetative cells move toward the root surface of *Arabidopsis thaliana*, where they form distinct biofilms consisting of a few layers of vegetative cells [14]–[16]. These observations prompted us to screen for mutants impaired in swarming motility and biofilm formation as likely preconditions for successful colonization and PGP.

We screened approximately 6,000 colonies from the transposon library for being impaired in swarming motility on swarming agar plates [21]. One mutant was found to be impaired in swarming. The chromosomal DNA of the mutant was used in an inverse PCR that amplified the chromosomal DNA abutting the transposon's ITR as described above. The amplified DNA was sequenced, and the sequence was identified as being an insertion within the *degU* gene sequence (Table 3). The swarming motility was restored in the *degU* Tn insertion mutant by transformation with wild-type chromosomal DNA. In addition, the wild-type FZB42 did not swarm when transformed with the *degU* mutant gene (Fig. S4A).

Screening for biofilm formation was performed in liquid MSgg medium. Under these conditions, the FZB42 wild-type develops aerial structures of white color while the previously colorless MSgg medium becomes slightly brown [16]. Again, the mutant clone harboring a *degU*::TnYLB-1 insertion was found impaired in biofilm formation. The mutant formed small structures that appeared to swim in the medium as isolated islands when grown in standing liquid MSgg medium, which remained colorless. Complementation of the *degU* mutant with the intact *degU* gene from FZB42 restored the ability of the mutant to form a biofilm in MSgg medium. By contrast, retransformation of the *degU*::TnYLB-1 fragment into the wild-type of *B. amyloliquefaciens* FZB42 resulted in transformants impaired in biofilm production bearing a phenotype similar to the *degU* mutant (Fig. S4B, Fig. S5).

Besides the *degU* insertion, we detected, out of 6,000 clones, two other mutant strains impaired in biofilm formation by screening in MSgg medium. They were identified as harboring insertions in the *pabA*, and the *yusV* gene, respectively (Table 3). The translated *yusV* gene sequence is similar to iron(III) dicitrate transport permease. The *pabA*::TnYLB-1 insertion displayed an unstructured layer of surface-attached cells characterized by flat and thin pellicles forming granules. The medium did not turn slightly brown. The *yusV*::TnYLB-1 insertion mutant formed pellicles with a flat and thin surface structure. Complementation of the *yusV* gene insertion by the intact wild-type gene restored the ability to produce standard biofilms. In the case of *pabA* it was possible to restore ability to form biofilms by adding *para*-amino benzoic acid (1 mM), which corroborates that the inability to form an intact biofilm is associated to a nutritional deficiency that negatively affected bacterial growth (Fig. S6).

### TnYLB-1 insertion mutants impaired in plant-growth-promotion

**Figure 1:**
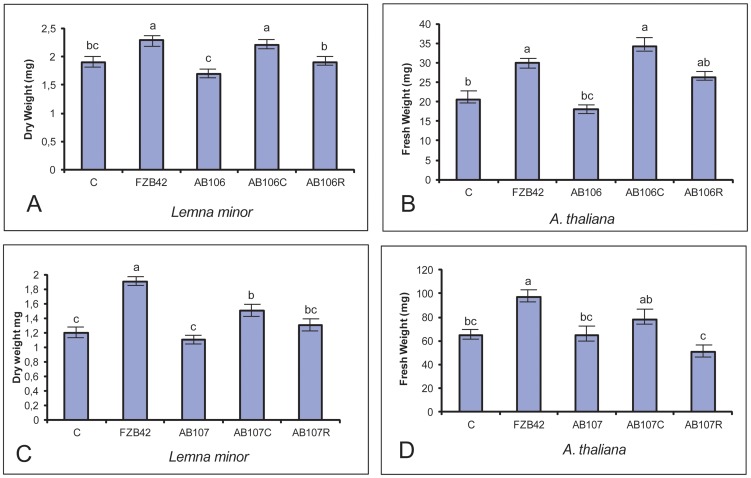
Effect on plant growth by mutant strains impaired in plant-growth-promotion. The mean dry or fresh weights obtained in four independent experiments including standard error were presented. Different letters indicate means that differ significantly (P≤ 0.05). Effect of AB106 (Δ*nfrA*) on growth of *L. minor* (A) and *A. thaliana* (B). C, control without bacteria, FZB42, control with FZB42, AB106, effect of AB106 (Δ*nfrA*), AB106C, effect of AB106 complemented by the *nfrA* wild-type gene (see Methods), AB106R, effect of FZB42 transformed with chromosomal DNA prepared from AB106 (Δ*nfrA*). Effect of AB107 (Δ*RBAM_17410*) on growth of *L. minor* (C) and *A. thaliana* (D). C, control without bacteria, FZB42, control with FZB42, AB107, effect of AB107 (Δ*RBAM_17410*), AB107C, effect of AB107 complemented by the *RBAM_17410* wild-type gene (see Materials and Methods), AB107R, effect of FZB42 transformed with chromosomal DNA prepared from AB107 (Δ*RBAM_17410*).

We searched for mutants impaired in plant-growth-promotion by using the *Lemna minor* biosystem. Inoculation of *L. minor* with FZB42 led to an increase of 20 to 30% in plant biomass (Fig. 1). Following random mutagenesis of *B. amyloliquefaciens* FZB42 with transposon TnYLB-1, three, out of the 3,000 clones investigated, were unable to exert the same effect. We identified the sites of the TnYLB-1 insertions by sequencing of the inverse PCR products as described in Materials and Methods. Genes affecting plant-growth-promotion were *nfrA* and *RBAM_017410*. The clone AB108 harbored an insertion outside of the coding region, within the *abrB* upstream region, and was excluded from further analysis (Table 3).

In the duckweed (*L. minor*) biotest system, the *nfrA*::TNYLB-1 insertion mutant (AB106) showed a reduction of more than 20% (p<0.05) in plant dry weight compared to the increase conferred by the wild-type. Complementation of TnYLB-1::*nfrA* by the *nfrA* wild-type gene restored the plant-growth promoting phenotype whilst the direct introduction of the TnYLB-1::*nfrA* insertion into the FZB42 wild-type *via* transformation with chromosomal DNA isolated from AB106 (Fig. S7) yielded reduced plant-growth promoting activity (Fig. 1A).

In addition, we examined the effect of the *nfrA* mutation on bacterial PGP in *Arabidopsis thaliana*. To exclude other, possibly interfering, factors, we applied a gnotobiotic system (see Materials and Methods). As in the *Lemna* system, a dramatic reduction of PGP, corresponding to that of the untreated control, was observed. Complementation of the *nfrA* mutant with the wild-type gene and retransformation of FZB42 with the TnYLB-1::*nfrA* gene yielded the expected results, corroborating those we had obtained with the *Lemna* system (Fig. 1 B).

Insertion of TnYLB-1 into the *RBAM_017410* gene, whose function is still unknown, resulted in a complete loss of the PGP effect in the *Lemna* system. The introduction of the *RBAM_017410* wild-type gene (Fig. S8) restored, at least in part, the PGP effect, whilst transformation of FZB42 with the mutant gene reduced the PGP effect exerted by the FZB42 wild-type. The *A. thaliana* system provided similar results (Fig. 1).

Taken together, the results obtained in two different gnotobiotic systems, *Lemna minor* and *Arabidopsis thaliana*, suggest that disruption of the genes *nfrA* and *RBAM_017410* by TnYLB-1 led to a loss in plant growth promoting activity. To elucidate mechanisms involved in plant-growth-promotion, we compared the growth at lettuce roots of the mutant strains impaired in motility, biofilm formation, and plant-growth-promotion with that of the wild-type FZB42.

### The *yusV, pabA, degU*, and *nfrA* mutants, but not the *RBAM_017410* mutant, were impaired in their ability to colonize lettuce roots

**Figure 2:**
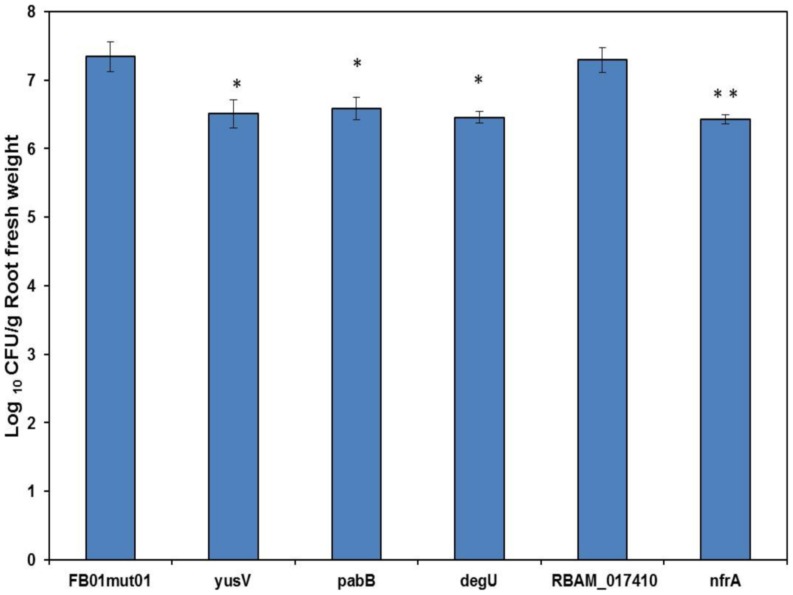
Rate of lettuce root colonization by gfp-labelled FZB42 (FB01mut01) and different TnYLB-1 insertion mutants (listed in **Table 1**). The data represent means of CFU obtained from three individual plants and standard deviations. Significant differences in means as obtained by two-tailed Student's *t* test are indicated by * (P≤ 0.05) and ** (P≤ 0.005).

In addition to the model systems, *Lemna minor* and *Arabidopsis thaliana*, we investigated root colonizing in a representative vegetable, *Lactuca sativa*. The number of CFU/g root fresh weight was determined one week after applying *Bacillus* spores on lettuce seedlings (Materials and Methods). Except for the *RBAM_017410* insertion mutant, all other mutant strains had approximately one order of magnitude reduced ability to colonize lettuce roots compared to the wild-type (Fig. 2), and it remains questionable whether a number of 10^6^ cfu/g root reflects active colonisation by that mutants, since a high concentration of spores (10^7^–10^8^/mL suspension) has been previously used to inoculate the lettuce roots (Materials and Methods). The reduced ability to grow at plant roots is remarkable, since the *nfrA*, *pabA*, *degU*, and *RBAM_017410* mutant strains grew as the wild-type FZB42, when cultivated under laboratory conditions in Luria broth (LB) medium. The mutant strain harboring a disrupted *yusV* gene was impaired in its growth in LB medium (Fig. S9), which suggests that in the case of *yusV* the reduced ability to colonize lettuce roots was due to a general growth defect in this mutant strain.

### The fate in colonizing lettuce roots by FZB42 in a chemically defined model system

**Figure 3:**
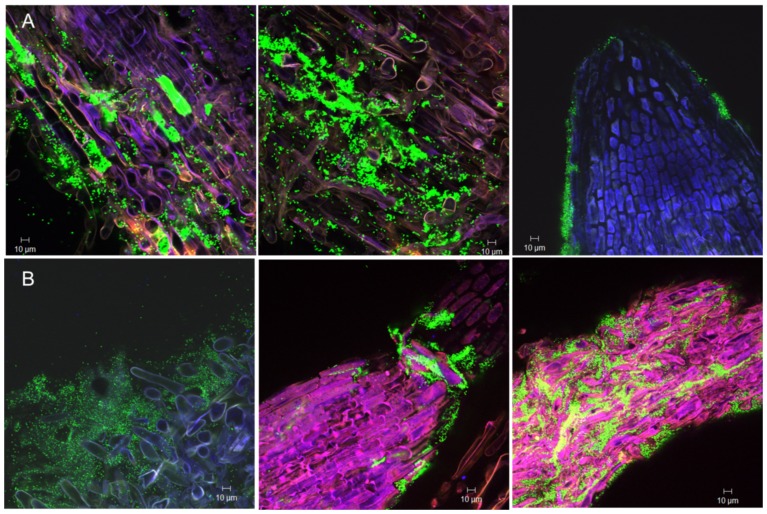
CLSM picture showing colonization of FB01mut01 on lettuce roots. A: One week after inoculation on epidermal cells (left), root hairs (middle) and root tip (right). B: Two weeks after inoculation on roots hairs (left and middle) and four weeks after inoculation of the primary root tip (right).

Two weeks after inoculation with the GFP-tagged *B. amyloliquefaciens* FZB42 FB01mut1 [14], CLSM showed that the epidermal layer of the lettuce root surface was colonized by FB01mut1. Conspicuous biofilm-like colonies occurred on the primary roots and on the base of emerging lateral roots. Bacterial colonization occurred mainly on primary roots and root hairs, as well as on root tips and adjacent border cells (Fig. 3). Occurrence of labelled bacteria decreased towards the root tips of the lateral roots, and no colonization of the finer roots could be observed. Z-stack pictures obtained by CLSM showed that there was no endophytic colonization of the roots (results not shown).

### Colonization patterns of TnYLB-1 insertion mutants

**Figure 4:**
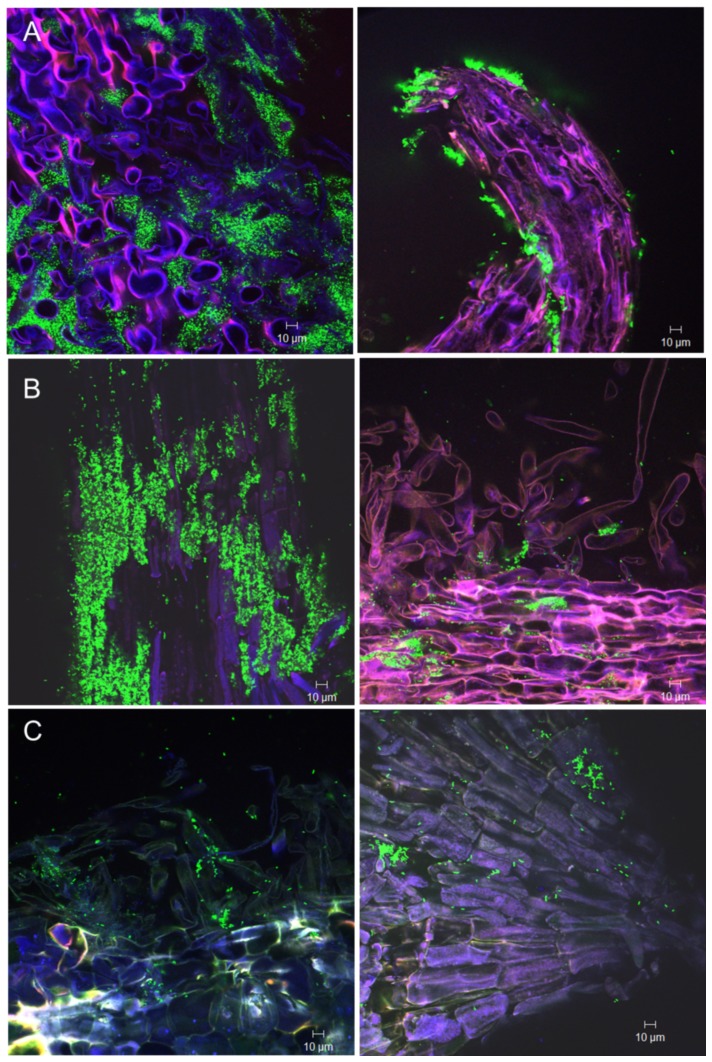
Colonization of lettuce roots by mutants impaired in biofilm formation. A: Mutant *yusV* after one week (on primary root surface, left) and two weeks (on lateral roots, right) of inoculation. B: Mutant *pabA* on primary root surface, one week (left) and two weeks (right) after inoculation. C: Mutant *degU* on primary root surface after one week and two weeks of inoculation.

The GFP-tagged *yusV* and *pabA* mutants grew similar during colonizing lettuce roots (Fig. 2). They colonized the surface of primary roots, but colonization of root hairs was reduced relative to the wild-type. In addition, the *yusV* transposon mutant colonized border cells of primary root tips (Fig. 4A and B). However, both mutant strains exhibited distinct phenotypes compared to the wild type during growth in Luria broth (LB). Whilst *pabA* was growing similarly to the wild-type, the *yusV* insertion mutant showed reduced growth, not exceeding an optical density at 600 nanometers (OD600) of 1.0 (Fig. S9, see previous section). The *degU* insertion mutant also diminished its general ability to colonize lettuce roots, being found scarcely on the primary root surface (Fig 4C). Nevertheless, its growth was indistinct from the wild-type when cultivated in LB medium under laboratory conditions (Fig. S9).

**Figure 5:**
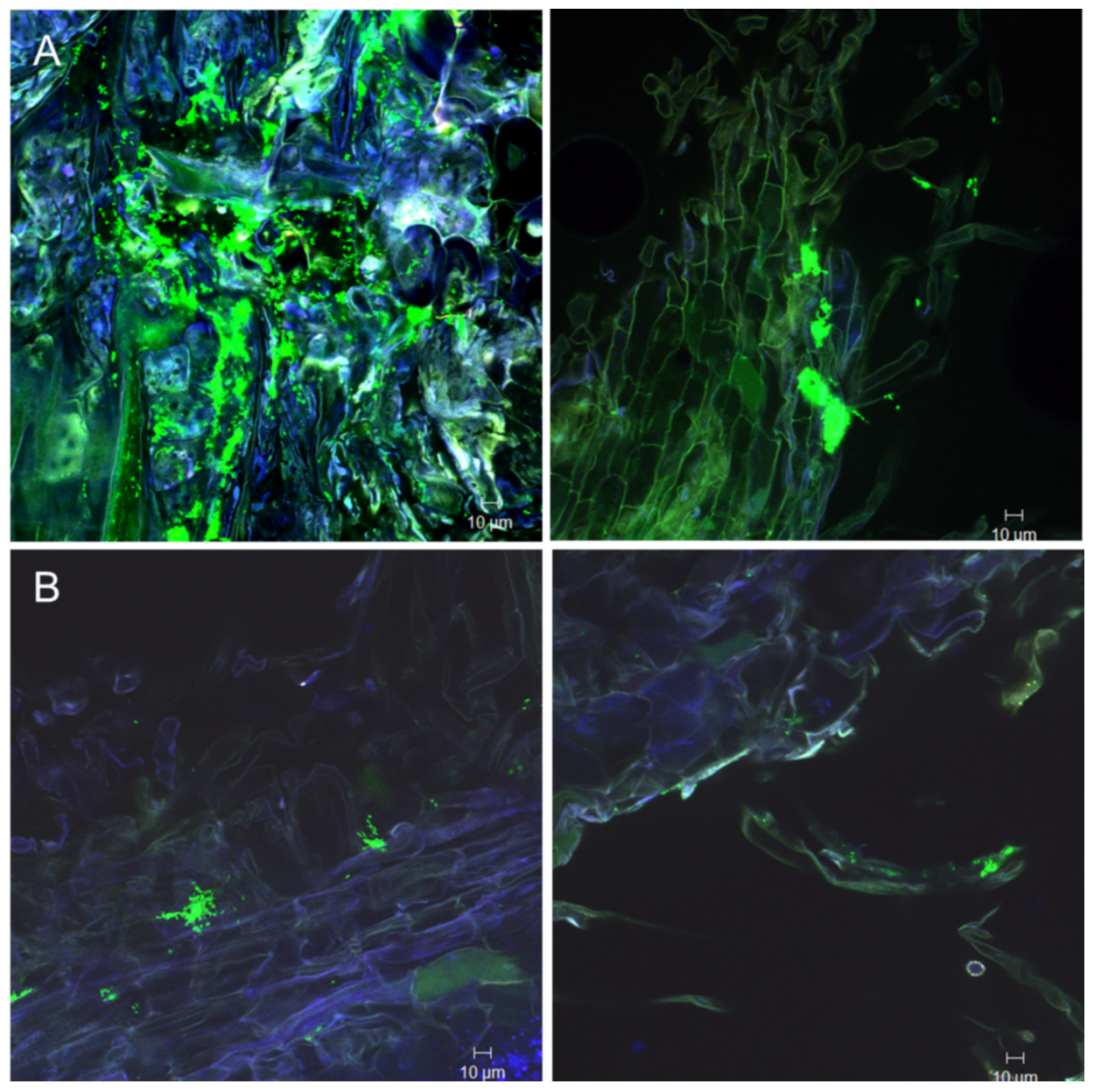
Colonization of lettuce roots by gfp-expressing mutants impaired in plant-growth-promotion. A: Mutant AB4 (*RBAM_017410*::TnYLB-1) after one week (on root hairs, left), and two weeks (on primary root surface, right) of inoculation. B: Mutant AB2 (*nfrA*::TnYLB-1) after one week (left) and two weeks (right) of inoculation.

**Figure 6:**
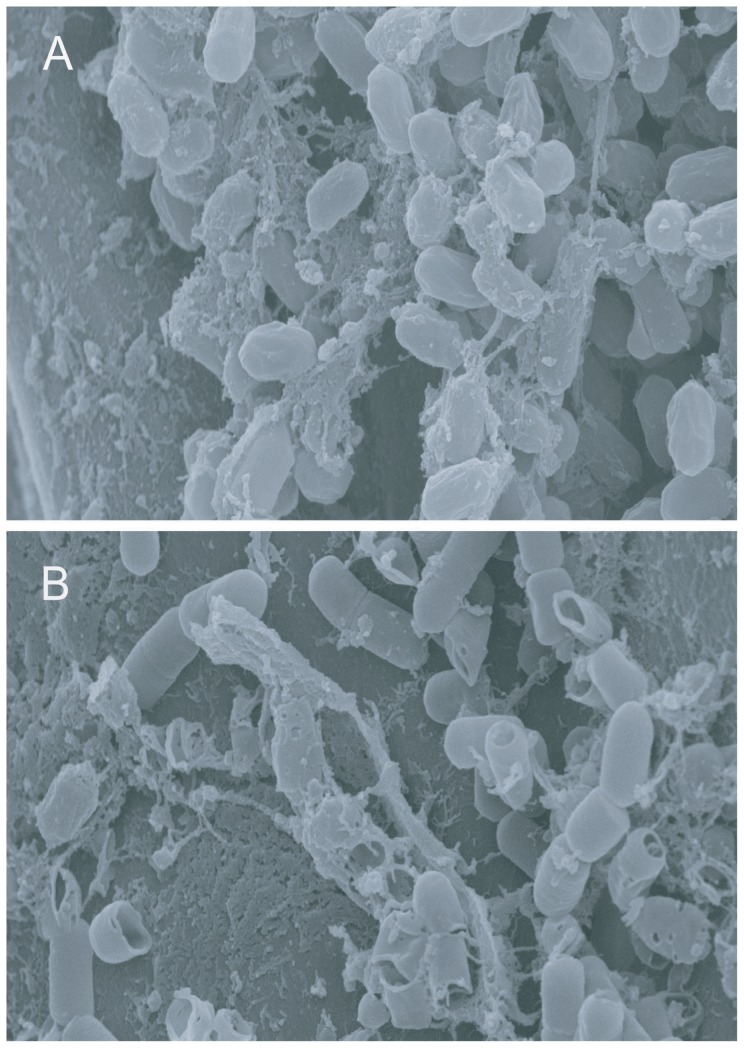
Scanning electron microscopy of FZB42 (A) and the *nfrA* mutant strain AB106 (B) colonizing *Arabidopsis* roots. The images were taken eight days after bacterial inoculation. The empty cell envelopes in the *nfrA* mutant strain (Fig. 6B) are clearly visible.

The two GFP labeled mutants impaired in PGP, AB2 (*nfrA*) and AB4 (*RBAM_017410*), showed a different colonization pattern on lettuce roots when compared to the wild-type (Table 4). The *RBAM_017410* mutant colonized efficiently the primary lettuce root surface, including root hairs. However, it was unable to colonize root tips or border cells (Fig. 5A), although no differences to the wild-type were found when grown under laboratory conditions in liquid culture (Fig. S9). The *nfrA* mutant AB2 was impaired in colonizing thelettuce roots (Fig. 2), and only a few fluorescent spots could be sporadically observed two weeks after inoculation with the *gfp* labelled mutant by CFM (Fig. 5B). By contrast, the *nfrA* mutant did not exhibit impaired growth under laboratory conditions (Fig. S9), biofilm formation and swarming motility in the agar screening tests (Table 4). This suggests that the observed growth limitation in the *nfrA* knock-out mutant was likely due to the plant response, and not to any general growth effect. Scanning electron microscopy performed with *Arabidopsis* roots treated with wild-type FZB42 (Fig. 6A) and the *nfrA* mutant (Fig. 6B) revealed the presence of numerous mutant cells on the root surface. However, while the wild-type cells remained intact, mutant cells appeared heavily damaged, and many empty cell debris were visible, suggesting that the nfrA mutant is not protected against the plant response reaction during colonization.

**Table 4:** Features of the transposon mutant strains.

Feature	FZB42	*degU*	*yusV*	*pabA*	*nfrA*	*RBAM_017410*
Growth in LB	**+**	**+**	**(+)**	**+**	**+**	**+**
Swarming	**+**	**-**	**+**	**+**	**+**	**+**
Biofilm	**+**	**-**	**-**	**-** 1	**+**	**+**
Root colonization^2^	**+**	**(+)**	**(+)**	**(+)**	**(+)**	**+**
Main root	**+**	**+**	**+**	**+**	**-**	**+**
Root tip	**+**	**-**	**-**	**-**	**-**	**-**
Border cells	**+**	**-**	**+**	**-**	**-**	**(+)**
Lateral roots	**+**	**-**	**+**	**+**	**-**	**+**
PGP	**+**	-	n.d.	n.d.	**-**	**-**
IAA (ng/ml)^3^						
−Trp	1.47±0.18	1.13±0.24	n.d.	n.d.	0.90±0.08	2.29±0.45
+Trp	15.9±2.8	26.4±4.35	n.d.	n.d.	16.0±3.6	24.2±9.2

1 biofilm formation restored by adding of *p*-amino benzoic acid

2 root colonization was performed in monoaxenic *Lactuca sativa* test system (see Materials and Methods and Fig. 2–4)

3 mean value including standard deviation calculated from six independent experiments in absence (-Trp) and presence (+Trp) of tryptophan (see Materials and Methods).

Colonization and other features were indicated as present +; reduced (+), and absent -. ‘n.d.’ stands for not determined. PGP: plant growth promotion.

### Reduction of plant-growth-promotion by mutant strains is not due to decreased 3-IAA production

To exclude that an already known mechanism is involved in the plant-growth-promoting effect exerted by *nfrA* and other mutant gene products described here, synthesis of the plant growth hormone indole-3-acetic acid (IAA) was determined. Using high-performance liquid chromatography (HPLC), and gas-chromatography-mass spectrometry (GC-MS) we have detected previously a tryptophan-dependent synthesis of indole-3-acetic acid (IAA) by FZB42^T^ [9]. Here, we corroborated this result using an alternative method, liquid-chromatography-mass spectrometry/mass spectrometry (see Materials and Methods). In general, IAA concentration in cultures grown in presence of tryptophan were found at least tenfold higher than in cultures grown in absence of tryptophan. Secretion of IAA in supernatants prepared from the *degU*, *nfrA*, and *RBAM_17410* insertion mutants were in the same range or slightly higher than that of the wild-type FZB42 (Table 4, Fig. S10). This suggests that the differences in plant growth promotion found in those mutants were not due to altered IAA synthesis.

## Discussion

Several mechanisms are involved in the growth promoting effect of rhizosphere bacteria, but successful root colonization is an essential initial step. In this study, we selected novel genes important for PGP, and examined the colonization of FZB42 and its mutants on lettuce roots using CLSM. We established a chemically defined axenic system with *Lactuca sativa* grown on quartz sand with Hoagland's medium. Our results demonstrated that FZB42 was an efficient lettuce root colonizer, and the pattern was similar to colonization of maize roots, mainly restricted to the primary root and adjacent root hairs rather than the root tips as in *Arabidopsis* [14]. The closed axenic system in this study cannot replace field or pot studies due to differences in plant growth and interactions with the microbial community. Nevertheless, this system is useful for studying colonization patterns to obtain an insight into the possible mechanisms involved in the plant growth promoting effect exerted by FZB42.

In order to generate mutants impaired in root colonization and plant-growth-promotion, we used a transposon library prepared from FZB42 with the mariner transposon *Himar1*. We examined three transposon insertion mutants defective in biofilm formation: *degU*, *yusV*, and *pabA*. The results obtained with these mutants, and the two mutants impaired in plant-growth-promotion (*nfrA* and *RBAM_017410*) are summarized in Table 4. It is evident that the products of the gene products examined in this study were acting on different levels of plant-bacteria interactions.

DegU is a known two-component response regulator involved in the control of extracellular macromolecule hydrolyzing enzyme synthesis [22] and competence [23] in *B. subtilis*. We have shown previously that DegU regulates the expression of the secondary metabolites bacillomycin D [24] and bacilysin [25] in FZB42. The data presented here suggest that DegU also acts as a positive regulator of genes involved in swarming motility and biofilm formation of FZB42 possibly through inhibition of the genes required for assembling flagella and complex colony architecture [26]. It is likely that DegU acts similarly in plant-associated *B. amyloliquefaciens* as in undomesticated strains of *B. subtilis*. DegU plays a pivotal role in controlling multicellular behavior [27]. It has been shown that both DegU and SwrAA are necessary to achieve full motility in undomesticated *B. subtilis* [28] and DegU initiates biofilm formation in the *B. subtilis* isolate RO-FF1 [29]. DegU∼P activates three targets. The first is poly-*γ*-glutamic acid, an extracellular polymer that can form part of the extracellular matrix of some isolates of *B. subtilis*, but not in NCIB 3610. This suggests that the specific requirements to form a multicellular population differ in a strain-dependent manner. The other two targets for DegU∼P in *B. subtilis* are the proteins YuaB and YvcA. The main role of DegU during biofilm formation is to drive indirectly the activation of transcription from the *yuaB* promoter. YuaB is a small, secreted protein that facilitates assembly of the biofilm matrix in ATCC 6051 and NCIB 3610 [30]. YvcA is a putative membrane-bound lipoprotein required for complex colony development in NCIB 3610 but not for pellicle formation in ATCC 6051 [27]. The genes involved in the multicellular behavior of FZB42 that are positively controlled by DegU remains to be identified. We found that the *yuaB* (*RBAM_028180*) gene, but not the *yvcA* gene, was present in the FZB42 genome. Our studies revealed that the ability of the *degU* mutant to colonize plant roots was diminished, suggesting that the formation of multicellular structures is an essential precondition for colonizing plant roots. Decreased cell densities of the *degU* mutant colonizing *Arabidopsis* seedlings relative to the wild type corroborated this finding. Production of 3-IAA was not reduced in the *degU* deficient strain excluding that DegU controls IAA synthesis in FZB42.

The other two mutants impaired in biofilm formation were *yusV::TnYLB-1* and *pabA::TnYLB-1*. The *yusV* gene product, a member of the Fur regulon, is induced under iron starvation. Iron acquisition in *B. subtilis* depends on intact YusV since *sfp^+^* strains, which are able to synthesize Fe-bacillibactin showed impaired growth under conditions of iron limitation when harboring a defect *yusV* gene. The YusV ATPase apparently functions as part of two different ABC uptake systems, the FeuABC bacillibactin siderophore, and the YfiZY/YfhA arthrobactin system [31]. Its reduced growth in LB (Fig. S9), containing between 8 and 10 µM iron [31], its reduced biofilm formation in synthetic medium, and root colonization are most likely due to a defect in iron uptake. The *pabA* gene encodes the subunit A of the *para*-aminobenzoic acid synthase (E.C. 2.6.1.85), an enzyme involved in synthesis of the vitamin folic acid [32]. We found that biofilm formation of the *pabA* mutant strain in synthetic media could be restored by adding *para*-aminobenzoic acid. Therefore, the inability to form a biofilm in a synthetic medium was probably associated with a growth restriction rather than an impaired trait specific to biofilm formation. Colonization of lettuce roots was diminished in both mutants, which suggests that insufficient amounts of the essential nutrients were available in the vicinity of plant root surfaces. We therefore presume that the *yusV* and *pabA* genes are not directly involved in biofilm formation.

The genes *nfrA* and *RBAM_017410* were directly involved in plant-growth-promotion. The *nfrA* mutant AB106 (Table 1) did not promote plant growth in the gnotobiotic systems established with *L. minor* and *A. thaliana*. The *nfrA* gene encodes an enzyme homologous to Spx-dependent-FMN-containing NADPH-linked nitro/flavin oxidoreductases, which is turned on under heat shock and oxidative stress conditions in *B. subtilis* [33]. Biochemical characterization of the NfrA protein in *Staphylococcus aureus* revealed that it is an NADPH-dependent FMN-containing oxidoreductase. However, its specific function remains to be elucidated [34]. The gene belongs to the class III heat shock genes and its transcription is induced in σ^D^-dependent manner at the onset of the stationary phase, and by heat stress from a σ^A^-promoter overlapping the σ^D^ promoter [35]. It has been reported that oxidative stress results in impaired biofilm formation of *B. subtilis* on *A. thaliana* roots [36]. DNA microarray analysis revealed that *nfrA* is induced by superoxide and H_2_O_2_, which indicates that the putative NADPH-dependent nitro/flavin reductase plays an important role in the oxidative stress response [37]. In this study, we found that the FZB42 *nfrA* mutant was inefficient in colonizing lettuce roots, although growth under laboratory conditions was not affected. SEM photographs of the *nfrA* mutant colonizing *Arabidopsis* roots demonstrated the presence of lysed cells possibly targeted by the plant defense responses during colonization (Fig. 6). At present, we speculate that the *nfrA* gene product plays a role in protecting FZB42 against the oxidative stress caused by the plant response during bacterial colonization. However, without experimental evidence for this hypothesis, further research is necessary to identify the molecular reason for the killing effect observed in the *nfrA* mutant cells attached at plant root surfaces.

The other gene identified in this screening as being important in PGP is *RBAM_017410*, which is located at nucleotides 1,809,412 to 1,809,597 in the FZB42 genome [6]. The gene encodes a 61 aa protein (YP_001421335.1) with unknown function containing five cysteine residues. It is a member of the prokaryotic RING finger family 1 characterized by a transmembrane domain. Prokaryotic RING fingers possess considerable functional diversity and not all of them are involved in ubiquitin-related functions [38]. Genome comparison revealed that, besides FZB42, orthologous genes are present in the genomes of only a few plant-associated *Bacillus* strains, such as AS43.3, EGD-AQ14, and 5B6, but were missing in most members of the *B. subtilis* species complex, including their plant-associated representatives. The *RBAM_017410* mutant strain AB107 was motile and able to form a biofilm in liquid MSgg medium. AB107 colonized lettuce roots grown in a gnotobiotic system consisting of sterilized quartz sand and 20 ml of Hoagland's solution as efficient as the wild-type. Interestingly, growth of AB107 in LB under laboratory conditions was not impaired. We hypothesize that, in contrast to the *nfrA* mutant, the product of the *RBAM_017410* gene is not linked with rhizosphere competence but supports directly PGP by a hitherto unknown mechanism.

Biofilm formation and efficient root colonization were reported as being important for successful PGP and biocontrol in *B. amyloliquefaciens* [39]. Here we could show that the gene products of *nfrA* and *RBAM_017141* were necessary for the beneficial effects exerted by FZB42 on plant growth. However, their mode of action seemed to be very different. We can rule out that solely growth-associated effects caused by nutrient deficiency or an altered ability to produce IAA are responsible for their effect on plant-growth-promotion. We hypothesize that the *nfrA* gene product, which is necessary for successful colonization of the competitive and highly selective plant rhizosphere, has a protecting function when the cells are exposed to oxidative stress caused by the plant response during bacterial colonizing of the root surface. By contrast, disruption of *RBAM_017410* did not affect multicellular behavior and ability to colonize lettuce roots, but this mutant was unable to support plant growth (Table 4). This shows that loss of the PGP effect is not always related to an inability to colonize plant roots and/or to form substantial biofilms.

## Supporting Information

Figure S1 Restriction digest of the plasmid DNA cut *by Eco*RI. Lanes 1–4: Plasmids isolated from transformed E. coli cells. Lane C: Plasmid pMarA digested with *Eco*RI.
(PPTX)

Figure S2 Construction and complementation of the *yusV* insertion mutant by the wild type *yusV* gene. A: Strategy for construction of the pUC18-D-*yusV* cassette. B: PCR product of the yusV gene. wild type FZB42 (lane1), *yusV* mutant (lane 2), complementation of *yusV* (lane 3) and retransformation of *yusV* (lane 4).
(PPTX)

Figure S3 **A**. Southern hybridization analysis of randomly chosen *B. amyloliquefaciens* FZB42 TnYLB-1 insertion mutants. Chromosomal DNA from *B. amyloliquefaciens* FZB42 (WT) and transposants (lanes 1–10) were digested with EcoRI and analyzed by Southern blotting using a hybridization probe specific for TnYLB-1. DNA fragment sizes (kbp) are indicated to the left and are based on DNA markers. **B.** PCR products of kanamycin gene, wild type FZB42 (lane 1) and the mutants (lane 2–20).
(PPTX)

Figure S4 Phenotypes of the *degU* mutant. Top (**A:Motility**): Phenotype of the degU insertion on swarming agar. Wild type FZB42 (A), *degU* insertion mutant (B), complementation by wt (C), and retransformation of the wt by the *degU* mutant gene (D). Bottom (**B: Biofilm**): Phenotype of degU insertion in biofilm formation checked in microtiter plates. Wild type FZB42 (A), *degU* mutant (B), complementation by wt (C), and retransformation of the wt by the *degU* mutant gene (D).
(PPTX)

Figure S5 Construction and complementation of the *deg*U insertion mutant by the wild type FZB42 *degU* gene. **A**: Strategy for construction of the pUC18-Δ*degU* cassette. **B**: PCR products of the *degU* gene. Wild type FZB42 (lane 1), *degU* TnYLB-1 insertion (lane 2), complementation by *degU* wild-type (lane 3), and replacement of the *degU* wild-type gene by the *degU::TnYLB-1* insertion (lane 4).
(PPTX)

Figure S6 Phenotype of biofilm formation in *yusV* and *pabA* insertion mutant strains. Top: *yusV*: FZB42 wild type (A), *yusV* mutant (B), complementation of *yusV* (C), and retransformation of *yusV* (D). Bottom: *pabA*: FZB42 wild type (A), *pabA* mutant (B), complementation of *pabA* (C), retransformation of *pabA* (D) and addition of 0.1 mM PABA (E).
(PPTX)

Figure S7 Construction and complementation of the *nfrA* insertion mutant by the wild type *nfrA* gene. A: Complementation of the *nrfA* mutant was done by amplifying the *nfrA* coding region plus 241 bp upstream sequences and 278 bp of downstream sequences using nfrA-dw-Eco88l and nfrA-up-ClaI primers, which contained Eco88l and Clal site, respectively. The fragment of *nfrA/*nfrA-dw-Eco88l/nfrA-up-ClaI (1269 bp) was cloned into linearized *ClaI/Eco88I* pUC18 plasmid which contained an *Amy* cassette (pVBF). B: PCR product of the *nfrA* gene. Wild type FZB42 (lane1), *nfrA* mutant (lane 2), complementation of*nfrA* (lane 3) and retransformation of *nfrA* (lane 4).
(PPTX)

Figure S8 Construction and complementation of the *RBAM_017410*::TnYLB-1 insertion mutant by the *RBAM_017410* wild type gene. A:Strategy for construction of the pUC18-Δ *RBAM_017410* cassette. B: PCR products of the *RBAM_017410* genes. Wild type FZB42 (lane1), *RBAM_017410::TnYLB-1* insertion mutant (lane 2), complementation of *RBAM_017410* (lane 3) and retransformation of *RBAM_017410* (lane 4).
(PPTX)

Figure S9 Growth of FZB42 and degU and nfrA mutant strains.
(PPTX)

Figure S10 IAA production of FZB42 and nrfA, degU, and RBAM_17410 (rBAM) mutant strains in absence (−T) and presence (+T) of Trp. Landy: medium control.
(PPTX)
