# Supplementary material for: Transposon Mutagenesis of the Plant-Associated Bacillus amyloliquefaciens ssp. plantarum FZB42 Revealed That the nfrA and RBAM17410 Genes Are Involved in Plant-Microbe-Interactions
Source: PLoS One. 2014 May 21;9(5):e98267. doi: 10.1371/journal.pone.0098267 (PMC4029887; doi:10.1371/journal.pone.0098267)
Supplement: Figure S1 — Restriction digest of the plasmid DNA cut by EcoRI. Lanes 1–4: Plasmids isolated from transformed E. coli cells. Lane C: Plasmid pMarA digested with EcoRI. (PPTX) [file pone.0098267.s001.pptx]

## Slide 1
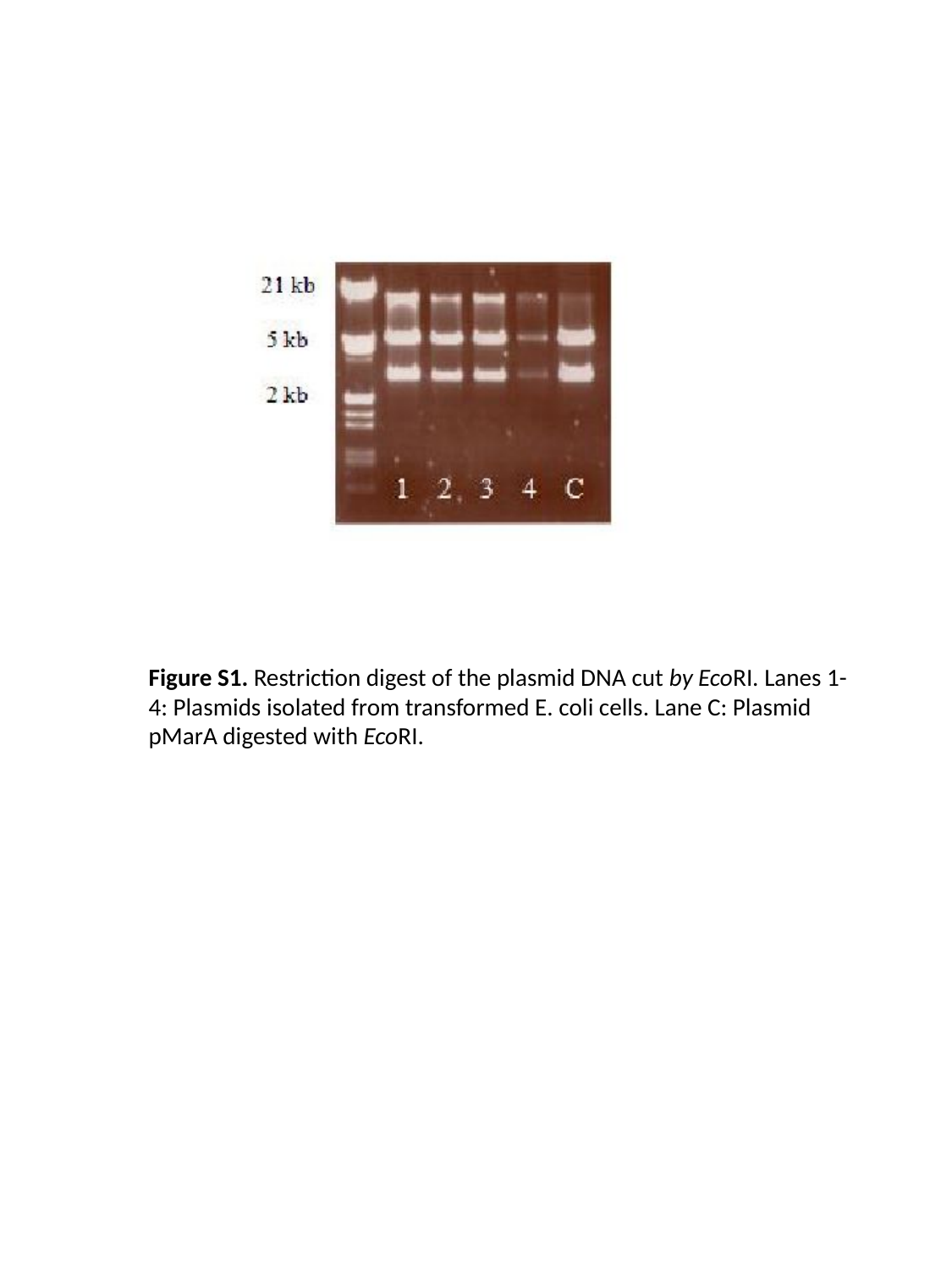

Figure S1. Restriction digest of the plasmid DNA cut by EcoRI. Lanes 1-4: Plasmids isolated from transformed E. coli cells. Lane C: Plasmid pMarA digested with EcoRI.
