# Supplementary material for: Transposon Mutagenesis of the Plant-Associated Bacillus amyloliquefaciens ssp. plantarum FZB42 Revealed That the nfrA and RBAM17410 Genes Are Involved in Plant-Microbe-Interactions
Source: PLoS One. 2014 May 21;9(5):e98267. doi: 10.1371/journal.pone.0098267 (PMC4029887; doi:10.1371/journal.pone.0098267)
Supplement: Figure S2 — Construction and complementation of the yusV insertion mutant by the wild type yusV gene. A: Strategy for construction of the pUC18-D-yusV cassette. B: PCR product of the yusV gene. wild type FZB42 (lane1), yusV mutant (lane 2), complementation of yusV (lane 3) and retransformation of yusV (lane 4). (PPTX) [file pone.0098267.s002.pptx]

## Slide 1
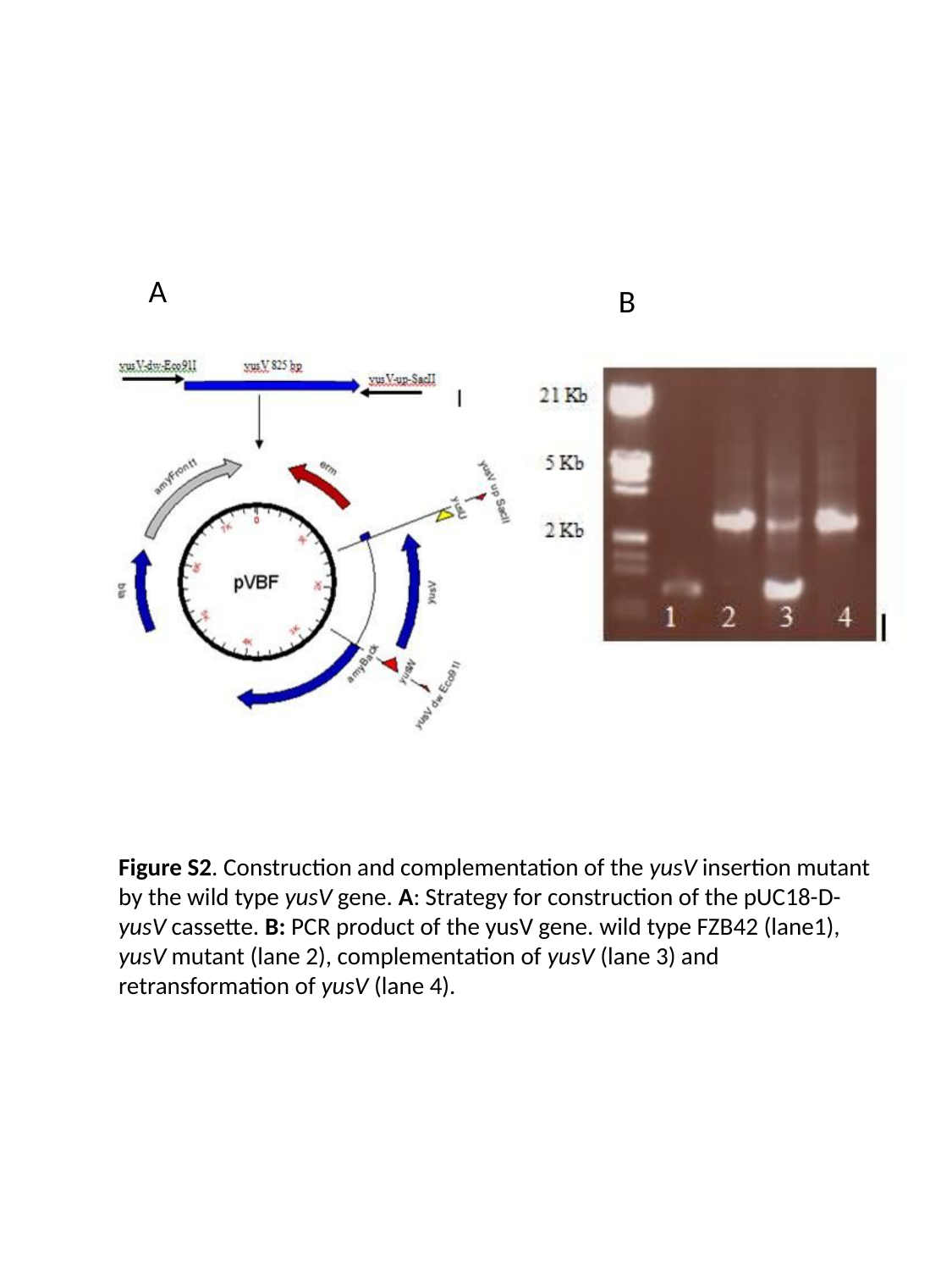

A
B
Figure S2. Construction and complementation of the yusV insertion mutant by the wild type yusV gene. A: Strategy for construction of the pUC18-D-yusV cassette. B: PCR product of the yusV gene. wild type FZB42 (lane1), yusV mutant (lane 2), complementation of yusV (lane 3) and retransformation of yusV (lane 4).
