# Supplementary material for: Transposon Mutagenesis of the Plant-Associated Bacillus amyloliquefaciens ssp. plantarum FZB42 Revealed That the nfrA and RBAM17410 Genes Are Involved in Plant-Microbe-Interactions
Source: PLoS One. 2014 May 21;9(5):e98267. doi: 10.1371/journal.pone.0098267 (PMC4029887; doi:10.1371/journal.pone.0098267)
Supplement: Figure S3 — A. Southern hybridization analysis of randomly chosen B. amyloliquefaciens FZB42 TnYLB-1 insertion mutants. Chromosomal DNA from B. amyloliquefaciens FZB42 (WT) and transposants (lanes 1–10) were digested with EcoRI and analyzed by Southern blotting using a hybridization probe specific for TnYLB-1. DNA fragment sizes (kbp) are indicated to the left and are based on DNA markers. B. PCR products of kanamycin gene, wild type FZB42 (lane 1) and the mutants (lane 2–20). (PPTX) [file pone.0098267.s003.pptx]

## Slide 1
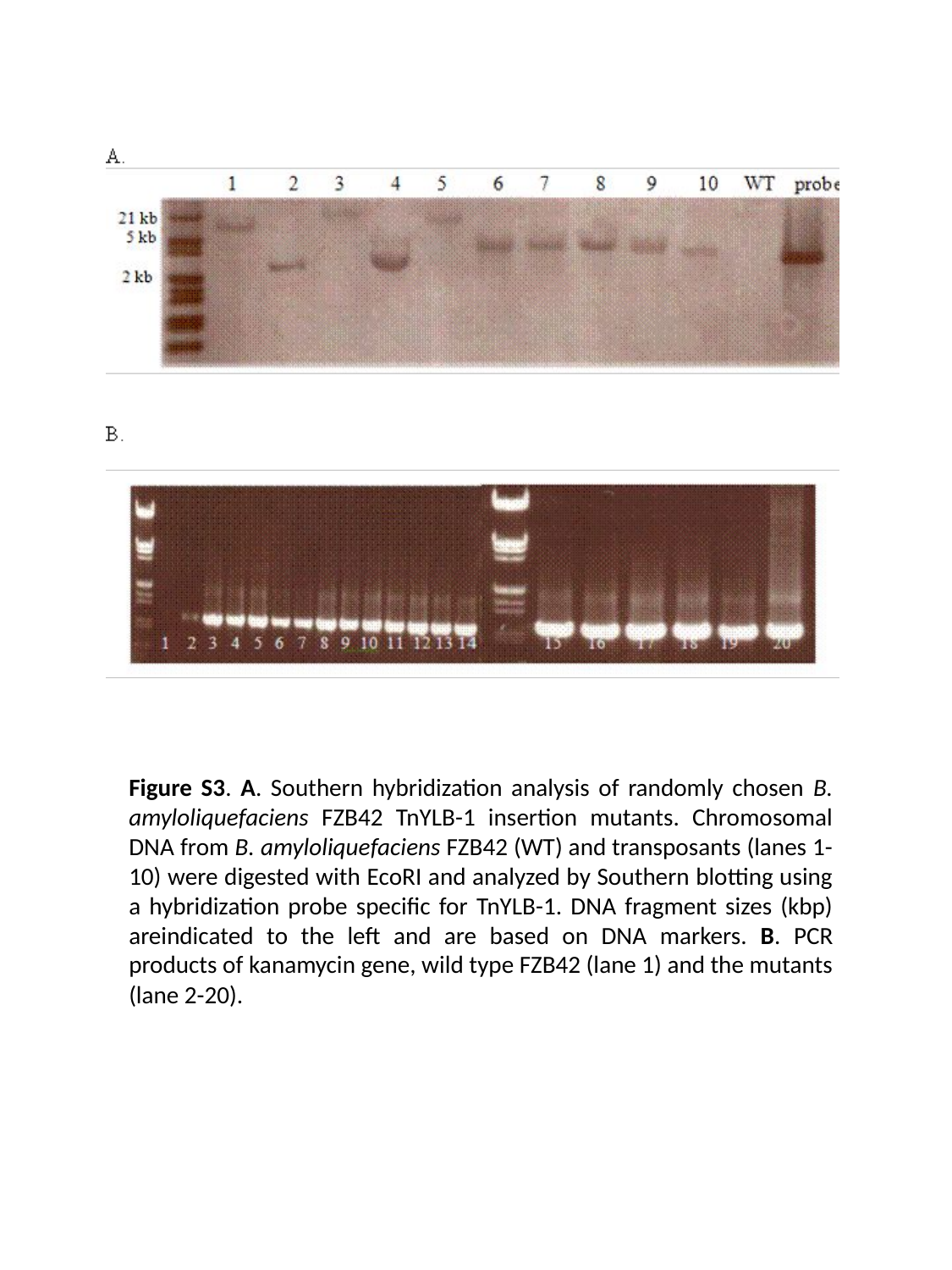

Figure S3. A. Southern hybridization analysis of randomly chosen B. amyloliquefaciens FZB42 TnYLB-1 insertion mutants. Chromosomal DNA from B. amyloliquefaciens FZB42 (WT) and transposants (lanes 1-10) were digested with EcoRI and analyzed by Southern blotting using a hybridization probe specific for TnYLB-1. DNA fragment sizes (kbp) areindicated to the left and are based on DNA markers. B. PCR products of kanamycin gene, wild type FZB42 (lane 1) and the mutants (lane 2-20).
