# Supplementary material for: Transposon Mutagenesis of the Plant-Associated Bacillus amyloliquefaciens ssp. plantarum FZB42 Revealed That the nfrA and RBAM17410 Genes Are Involved in Plant-Microbe-Interactions
Source: PLoS One. 2014 May 21;9(5):e98267. doi: 10.1371/journal.pone.0098267 (PMC4029887; doi:10.1371/journal.pone.0098267)
Supplement: Figure S4 — Phenotypes of the degU mutant. Top (A:Motility): Phenotype of the degU insertion on swarming agar. Wild type FZB42 (A), degU insertion mutant (B), complementation by wt (C), and retransformation of the wt by the degU mutant gene (D). Bottom (B: Biofilm): Phenotype of degU insertion in biofilm formation checked in microtiter plates. Wild type FZB42 (A), degU mutant (B), complementation by wt (C), and retransformation of the wt by the degU mutant gene (D). (PPTX) [file pone.0098267.s004.pptx]

## Slide 1
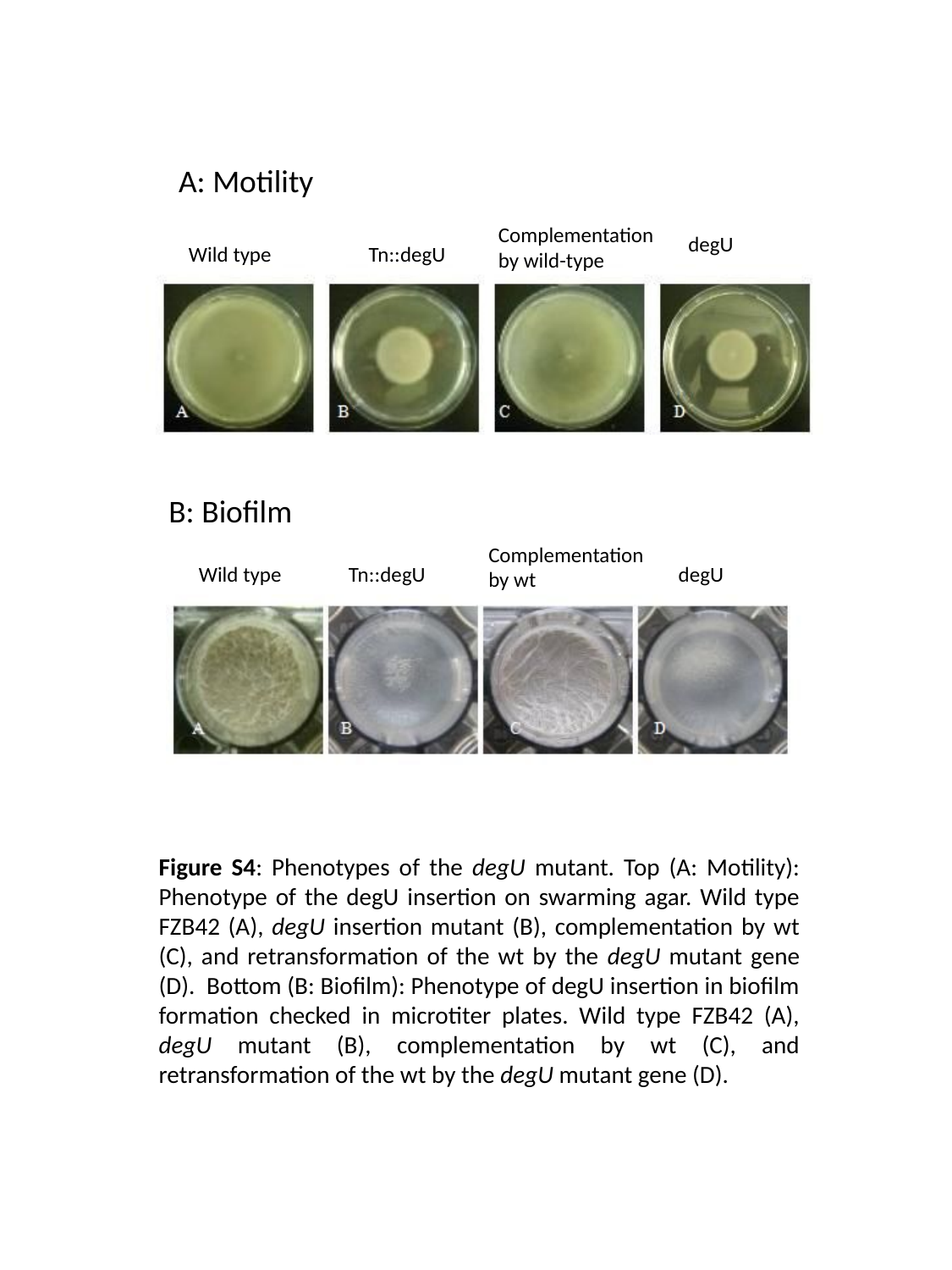

A: Motility
Complementation by wild-type
degU
Wild type
Tn::degU
B: Biofilm
Complementation by wt
Wild type
Tn::degU
degU
Figure S4: Phenotypes of the degU mutant. Top (A: Motility): Phenotype of the degU insertion on swarming agar. Wild type FZB42 (A), degU insertion mutant (B), complementation by wt (C), and retransformation of the wt by the degU mutant gene (D). Bottom (B: Biofilm): Phenotype of degU insertion in biofilm formation checked in microtiter plates. Wild type FZB42 (A), degU mutant (B), complementation by wt (C), and retransformation of the wt by the degU mutant gene (D).
