# Supplementary material for: Transposon Mutagenesis of the Plant-Associated Bacillus amyloliquefaciens ssp. plantarum FZB42 Revealed That the nfrA and RBAM17410 Genes Are Involved in Plant-Microbe-Interactions
Source: PLoS One. 2014 May 21;9(5):e98267. doi: 10.1371/journal.pone.0098267 (PMC4029887; doi:10.1371/journal.pone.0098267)
Supplement: Figure S5 — Construction and complementation of the degU insertion mutant by the wild type FZB42 degU gene. A: Strategy for construction of the pUC18-ΔdegU cassette. B: PCR products of the degU gene. Wild type FZB42 (lane 1), degU TnYLB-1 insertion (lane 2), complementation by degU wild-type (lane 3), and replacement of the degU wild-type gene by the degU::TnYLB-1 insertion (lane 4). (PPTX) [file pone.0098267.s005.pptx]

## Slide 1
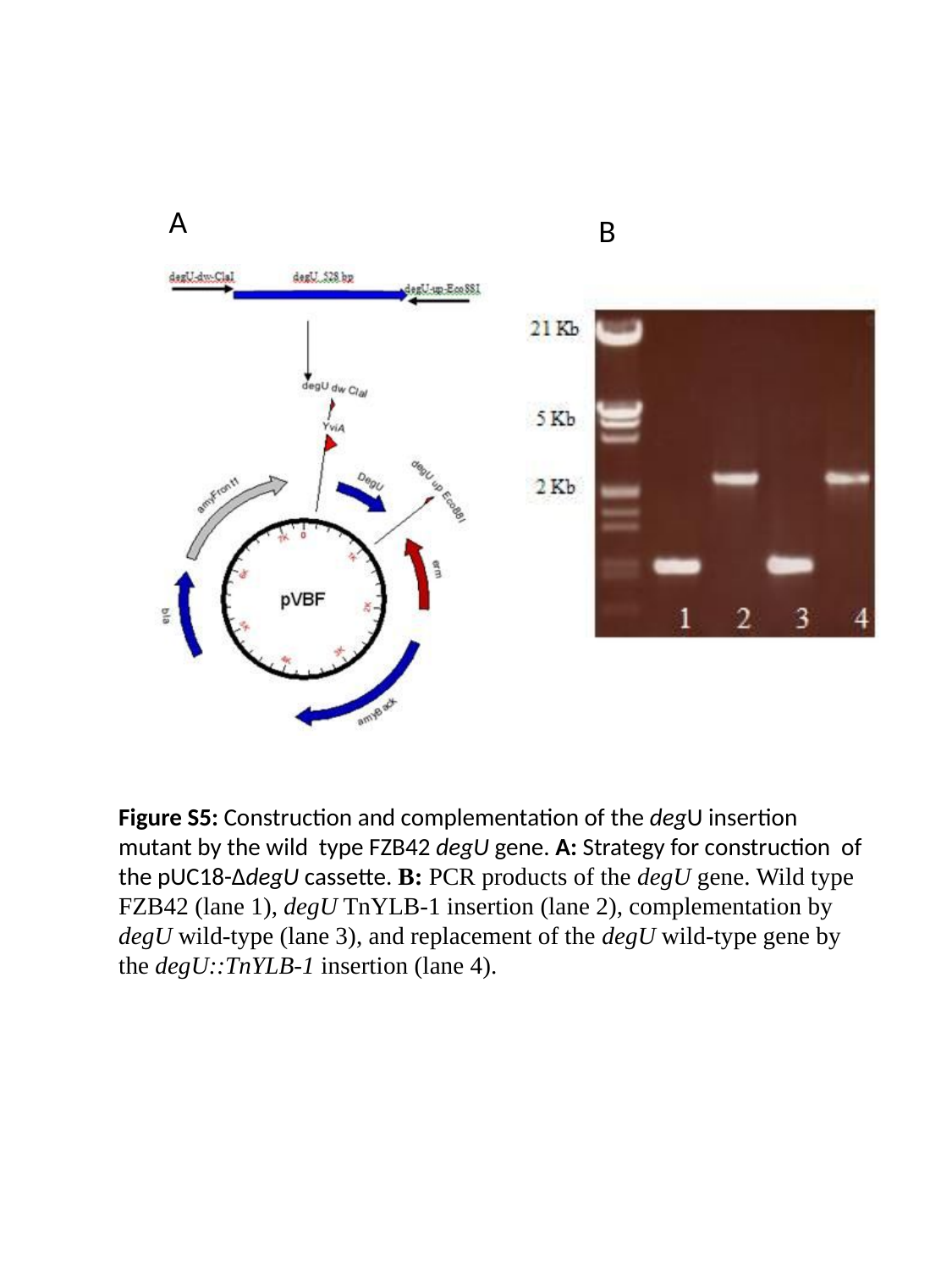

A
B
Figure S5: Construction and complementation of the degU insertion mutant by the wild type FZB42 degU gene. A: Strategy for construction of the pUC18-∆degU cassette. B: PCR products of the degU gene. Wild type FZB42 (lane 1), degU TnYLB-1 insertion (lane 2), complementation by degU wild-type (lane 3), and replacement of the degU wild-type gene by the degU::TnYLB-1 insertion (lane 4).
