# Supplementary material for: Transposon Mutagenesis of the Plant-Associated Bacillus amyloliquefaciens ssp. plantarum FZB42 Revealed That the nfrA and RBAM17410 Genes Are Involved in Plant-Microbe-Interactions
Source: PLoS One. 2014 May 21;9(5):e98267. doi: 10.1371/journal.pone.0098267 (PMC4029887; doi:10.1371/journal.pone.0098267)
Supplement: Figure S6 — Phenotype of biofilm formation in yusV and pabA insertion mutant strains. Top: yusV: FZB42 wild type (A), yusV mutant (B), complementation of yusV (C), and retransformation of yusV (D). Bottom: pabA: FZB42 wild type (A), pabA mutant (B), complementation of pabA (C), retransformation of pabA (D) and addition of 0.1 mM PABA (E). (PPTX) [file pone.0098267.s006.pptx]

## Slide 1
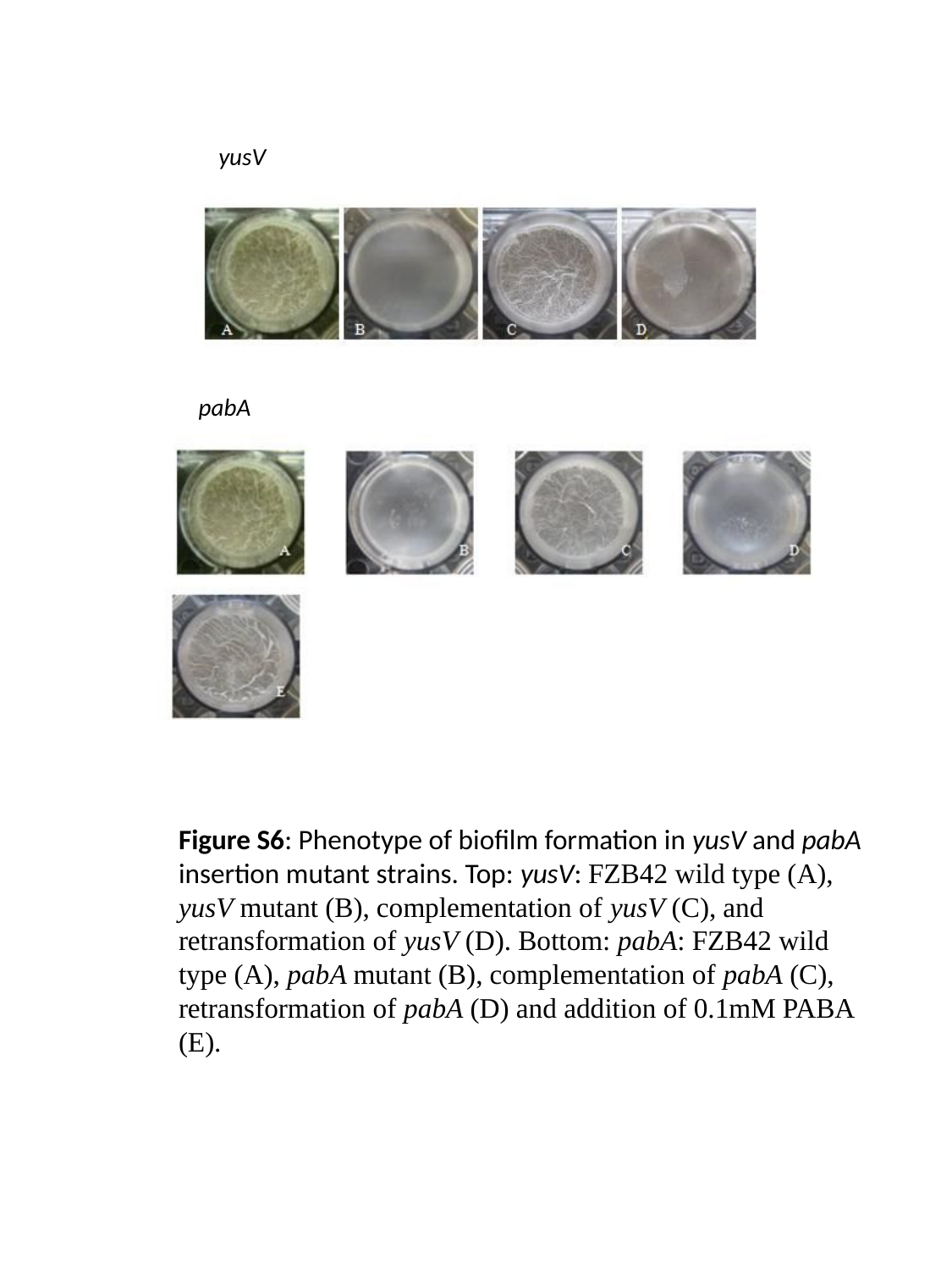

yusV
pabA
Figure S6: Phenotype of biofilm formation in yusV and pabA insertion mutant strains. Top: yusV: FZB42 wild type (A), yusV mutant (B), complementation of yusV (C), and retransformation of yusV (D). Bottom: pabA: FZB42 wild type (A), pabA mutant (B), complementation of pabA (C), retransformation of pabA (D) and addition of 0.1mM PABA (E).
