# Supplementary material for: Transposon Mutagenesis of the Plant-Associated Bacillus amyloliquefaciens ssp. plantarum FZB42 Revealed That the nfrA and RBAM17410 Genes Are Involved in Plant-Microbe-Interactions
Source: PLoS One. 2014 May 21;9(5):e98267. doi: 10.1371/journal.pone.0098267 (PMC4029887; doi:10.1371/journal.pone.0098267)
Supplement: Figure S7 — Construction and complementation of the nfrA insertion mutant by the wild type nfrA gene. A: Complementation of the nrfA mutant was done by amplifying the nfrA coding region plus 241 bp upstream sequences and 278 bp of downstream sequences using nfrA-dw-Eco88l and nfrA-up-ClaI primers, which contained Eco88l and Clal site, respectively. The fragment of nfrA/nfrA-dw-Eco88l/nfrA-up-ClaI (1269 bp) was cloned into linearized ClaI/Eco88I pUC18 plasmid which contained an Amy cassette (pVBF). B: PCR product of the nfrA gene. Wild type FZB42 (lane1), nfrA mutant (lane 2), complementation ofnfrA (lane 3) and retransformation of nfrA (lane 4). (PPTX) [file pone.0098267.s007.pptx]

## Slide 1
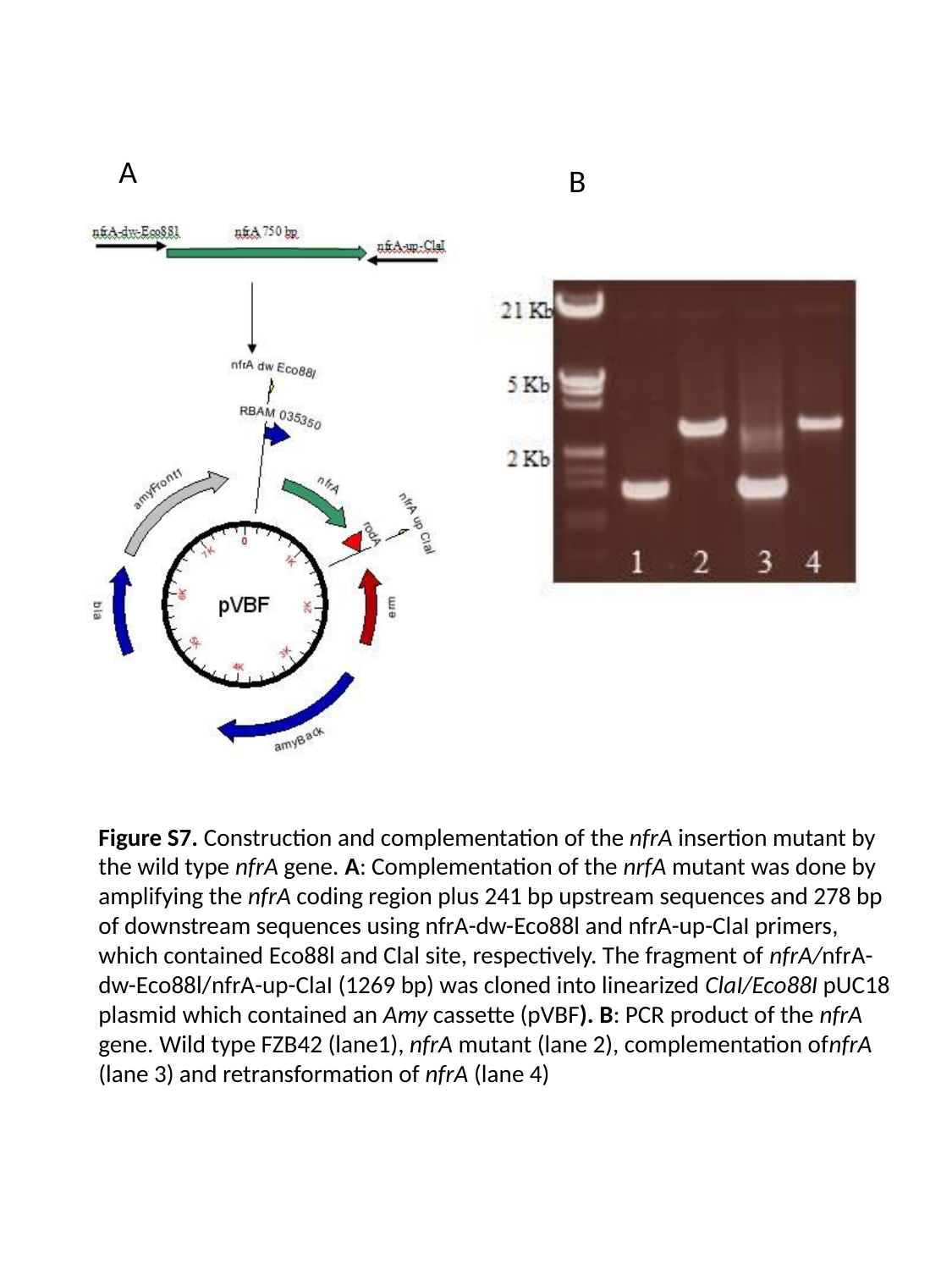

A
B
Figure S7. Construction and complementation of the nfrA insertion mutant by the wild type nfrA gene. A: Complementation of the nrfA mutant was done by amplifying the nfrA coding region plus 241 bp upstream sequences and 278 bp of downstream sequences using nfrA-dw-Eco88l and nfrA-up-ClaI primers, which contained Eco88l and Clal site, respectively. The fragment of nfrA/nfrA-dw-Eco88l/nfrA-up-ClaI (1269 bp) was cloned into linearized ClaI/Eco88I pUC18 plasmid which contained an Amy cassette (pVBF). B: PCR product of the nfrA gene. Wild type FZB42 (lane1), nfrA mutant (lane 2), complementation ofnfrA (lane 3) and retransformation of nfrA (lane 4)
