# Supplementary material for: Transposon Mutagenesis of the Plant-Associated Bacillus amyloliquefaciens ssp. plantarum FZB42 Revealed That the nfrA and RBAM17410 Genes Are Involved in Plant-Microbe-Interactions
Source: PLoS One. 2014 May 21;9(5):e98267. doi: 10.1371/journal.pone.0098267 (PMC4029887; doi:10.1371/journal.pone.0098267)
Supplement: Figure S8 — Construction and complementation of the RBAM_017410::TnYLB-1 insertion mutant by the RBAM_017410 wild type gene. A:Strategy for construction of the pUC18-Δ RBAM_017410 cassette. B: PCR products of the RBAM_017410 genes. Wild type FZB42 (lane1), RBAM_017410::TnYLB-1 insertion mutant (lane 2), complementation of RBAM_017410 (lane 3) and retransformation of RBAM_017410 (lane 4). (PPTX) [file pone.0098267.s008.pptx]

## Slide 1
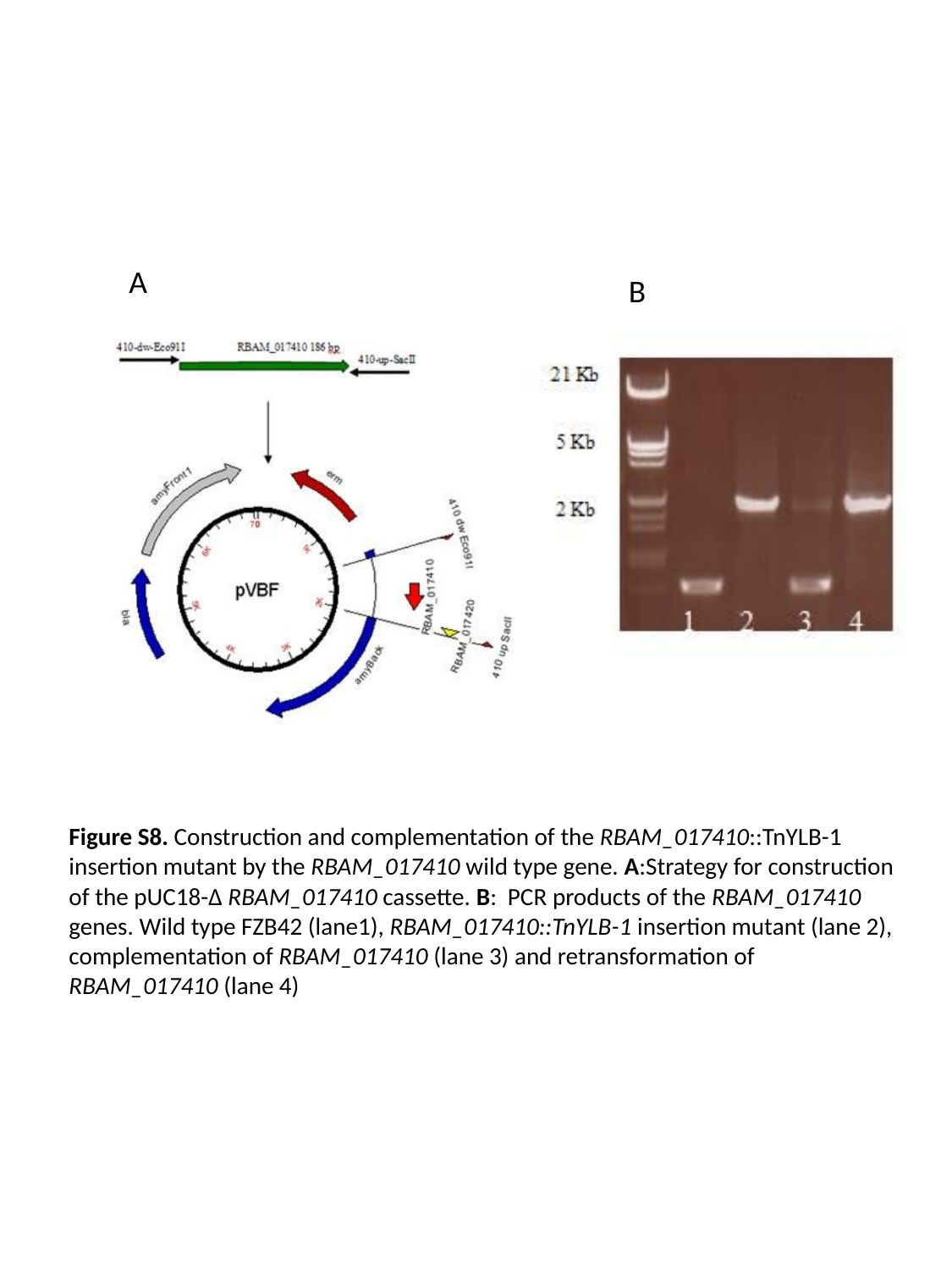

A
B
# Figure S8. Construction and complementation of the RBAM_017410::TnYLB-1 insertion mutant by the RBAM_017410 wild type gene. A:Strategy for construction of the pUC18-∆ RBAM_017410 cassette. B: PCR products of the RBAM_017410 genes. Wild type FZB42 (lane1), RBAM_017410::TnYLB-1 insertion mutant (lane 2), complementation of RBAM_017410 (lane 3) and retransformation of RBAM_017410 (lane 4)
