# Supplementary material for: Transposon Mutagenesis of the Plant-Associated Bacillus amyloliquefaciens ssp. plantarum FZB42 Revealed That the nfrA and RBAM17410 Genes Are Involved in Plant-Microbe-Interactions
Source: PLoS One. 2014 May 21;9(5):e98267. doi: 10.1371/journal.pone.0098267 (PMC4029887; doi:10.1371/journal.pone.0098267)
Supplement: Figure S9 — Growth of FZB42 and degU and nfrA mutant strains. (PPTX) [file pone.0098267.s009.pptx]

## Slide 1
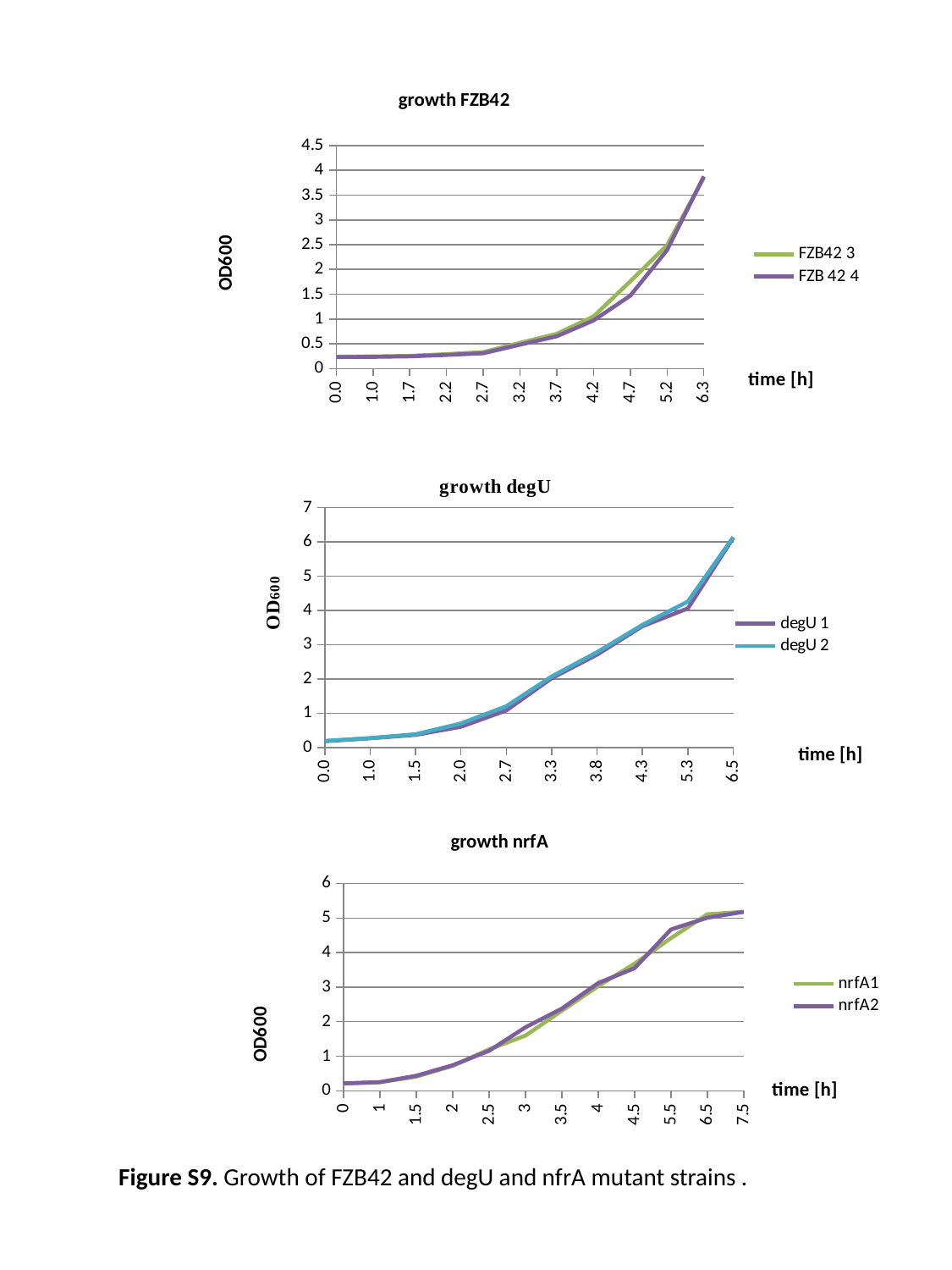

### Chart
| Category | FZB42 3 | FZB 42 4 |
|---|---|---|
| 0 | 0.23800000000000004 | 0.232 |
| 1 | 0.24400000000000008 | 0.23600000000000004 |
| 1.6666666666666667 | 0.256 | 0.24600000000000008 |
| 2.1666666666666665 | 0.29000000000000015 | 0.274 |
| 2.6666666666666665 | 0.33200000000000024 | 0.30800000000000016 |
| 3.1666666666666665 | 0.518 | 0.48200000000000015 |
| 3.6666666666666665 | 0.7000000000000003 | 0.6500000000000004 |
| 4.166666666666667 | 1.05 | 0.9700000000000003 |
| 4.666666666666667 | 1.76 | 1.47 |
| 5.166666666666667 | 2.4899999999999998 | 2.3899999999999997 |
| 6.3333333333333366 | 3.8499999999999988 | 3.88 |
### Chart
| Category | degU 1 | degU 2 |
|---|---|---|
| 0 | 0.18000000000000008 | 0.19000000000000009 |
| 1 | 0.266 | 0.272 |
| 1.5 | 0.3650000000000002 | 0.3840000000000002 |
| 2 | 0.6020000000000003 | 0.6990000000000005 |
| 2.6666666666666665 | 1.08 | 1.2 |
| 3.3333333333333335 | 2.02 | 2.07 |
| 3.8333333333333335 | 2.71 | 2.7800000000000002 |
| 4.3333333333333366 | 3.54 | 3.58 |
| 5.3333333333333366 | 4.06 | 4.26 |
| 6.5 | 6.119999999999997 | 6.14 |time [h]
### Chart
| Category | nrfA1 | nrfA2 |
|---|---|---|
| 0 | 0.21000000000000008 | 0.21600000000000008 |
| 1 | 0.24800000000000008 | 0.252 |
| 1.5 | 0.41200000000000014 | 0.43800000000000017 |
| 2 | 0.7240000000000003 | 0.7420000000000003 |
| 2.5 | 1.2 | 1.1599999999999993 |
| 3 | 1.6 | 1.84 |
| 3.5 | 2.3199999999999985 | 2.38 |
| 4 | 3.03 | 3.12 |
| 4.5 | 3.68 | 3.55 |
| 5.5 | 4.42 | 4.67 |
| 6.5 | 5.1099999999999985 | 5.01 |
| 7.5 | 5.18 | 5.18 |Figure S9. Growth of FZB42 and degU and nfrA mutant strains .

## Slide 2
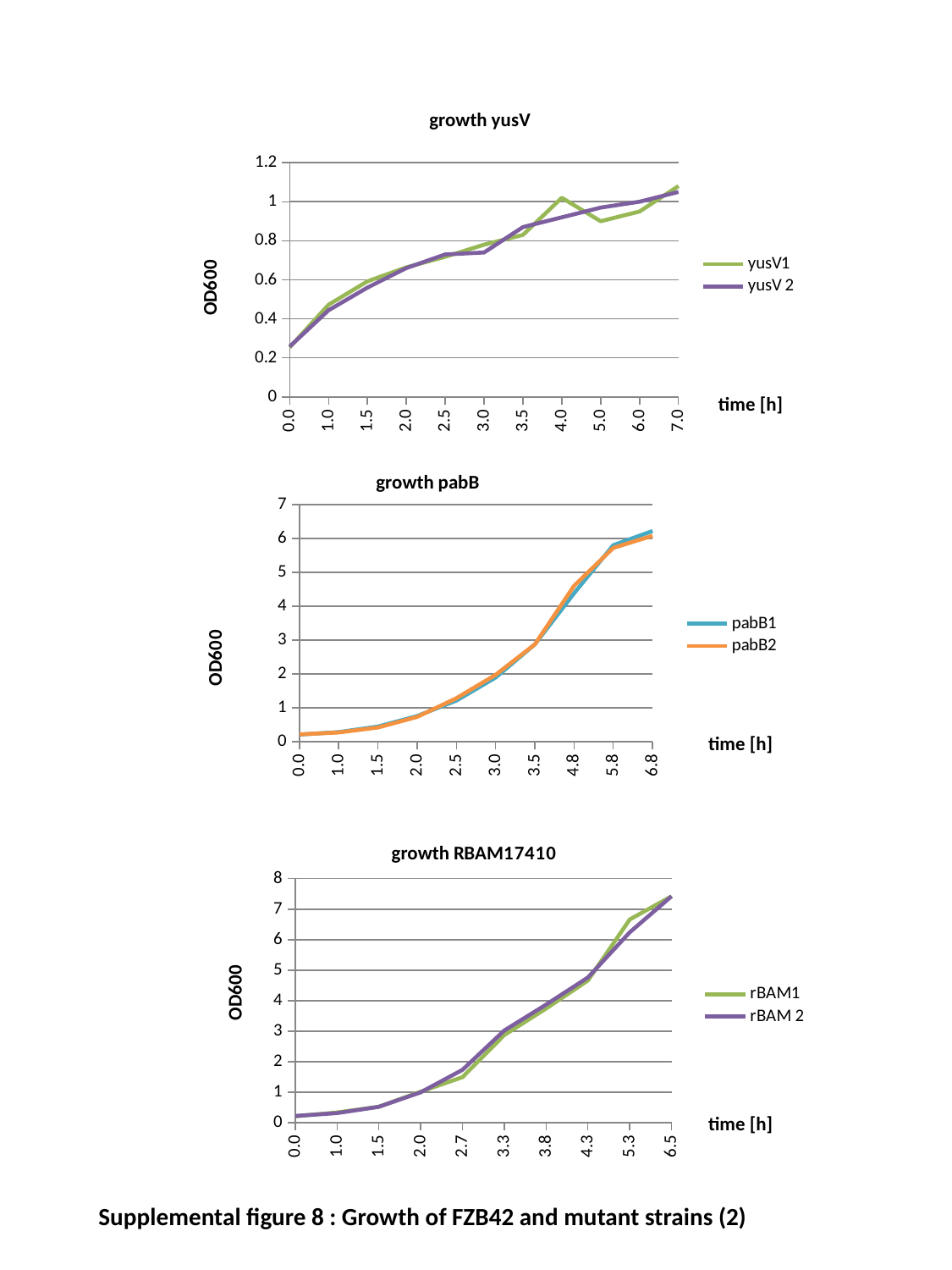

### Chart
| Category | yusV1 | yusV 2 |
|---|---|---|
| 0 | 0.254 | 0.258 |
| 1 | 0.47200000000000014 | 0.444 |
| 1.5 | 0.592 | 0.56 |
| 2 | 0.6640000000000005 | 0.6600000000000004 |
| 2.5 | 0.7180000000000003 | 0.7300000000000003 |
| 3 | 0.78 | 0.7400000000000003 |
| 3.5 | 0.8300000000000003 | 0.8700000000000003 |
| 4 | 1.02 | 0.92 |
| 5 | 0.9 | 0.9700000000000003 |
| 6 | 0.9500000000000003 | 1.0 |
| 7 | 1.08 | 1.05 |time [h]
### Chart
| Category | pabB1 | pabB2 |
|---|---|---|
| 0 | 0.20600000000000004 | 0.21200000000000008 |
| 1 | 0.2800000000000001 | 0.27 |
| 1.5 | 0.444 | 0.41600000000000015 |
| 2 | 0.7540000000000003 | 0.7280000000000003 |
| 2.5 | 1.21 | 1.28 |
| 3 | 1.8900000000000001 | 1.9700000000000006 |
| 3.5 | 2.8699999999999997 | 2.8699999999999997 |
| 4.75 | 4.38 | 4.6 |
| 5.75 | 5.8 | 5.72 |
| 6.75 | 6.22 | 6.08 |time [h]
### Chart
| Category | rBAM1 | rBAM 2 |
|---|---|---|
| 0 | 0.20800000000000007 | 0.21800000000000008 |
| 1 | 0.33200000000000024 | 0.31200000000000017 |
| 1.5 | 0.52 | 0.518 |
| 2 | 1.014 | 0.992 |
| 2.6666666666666665 | 1.49 | 1.73 |
| 3.3333333333333335 | 2.8699999999999997 | 3.02 |
| 3.8333333333333335 | 3.74 | 3.8699999999999997 |
| 4.3333333333333366 | 4.6599999999999975 | 4.76 |
| 5.3333333333333366 | 6.6599999999999975 | 6.24 |
| 6.5 | 7.42 | 7.42 |OD600
time [h]
Supplemental figure 8 : Growth of FZB42 and mutant strains (2)
