# Supplementary material for: Transposon Mutagenesis of the Plant-Associated Bacillus amyloliquefaciens ssp. plantarum FZB42 Revealed That the nfrA and RBAM17410 Genes Are Involved in Plant-Microbe-Interactions
Source: PLoS One. 2014 May 21;9(5):e98267. doi: 10.1371/journal.pone.0098267 (PMC4029887; doi:10.1371/journal.pone.0098267)
Supplement: Figure S10 — IAA production of FZB42 and nrfA, degU, and RBAM_17410 (rBAM) mutant strains in absence (−T) and presence (+T) of Trp. Landy: medium control. (PPTX) [file pone.0098267.s010.pptx]

## Slide 1
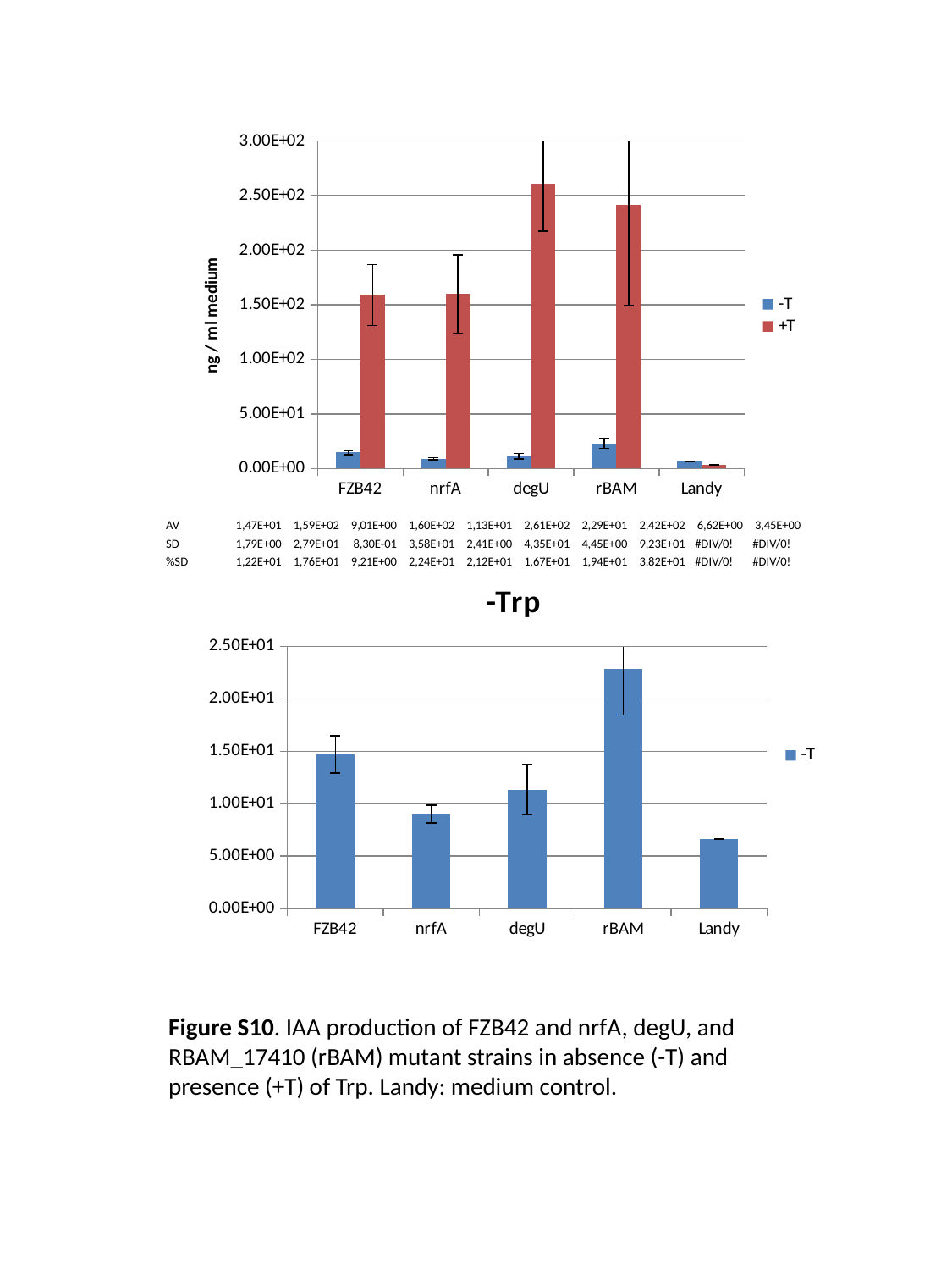

### Chart
| Category | | |
|---|---|---|
| FZB42 | 14.702395859997624 | 159.08808342106053 |
| nrfA | 9.008334295499482 | 159.9417435201652 |
| degU | 11.323834116598489 | 261.0862043296316 |
| rBAM | 22.89917356394677 | 241.70875866167015 |
| Landy | 6.622807017543858 | 3.4502923976608177 || AV | 1,47E+01 | 1,59E+02 | 9,01E+00 | 1,60E+02 | 1,13E+01 | 2,61E+02 | 2,29E+01 | 2,42E+02 | 6,62E+00 | 3,45E+00 |
| --- | --- | --- | --- | --- | --- | --- | --- | --- | --- | --- |
| SD | 1,79E+00 | 2,79E+01 | 8,30E-01 | 3,58E+01 | 2,41E+00 | 4,35E+01 | 4,45E+00 | 9,23E+01 | #DIV/0! | #DIV/0! |
| %SD | 1,22E+01 | 1,76E+01 | 9,21E+00 | 2,24E+01 | 2,12E+01 | 1,67E+01 | 1,94E+01 | 3,82E+01 | #DIV/0! | #DIV/0! |
### Chart: -Trp
| Category | |
|---|---|
| FZB42 | 14.702395859997624 |
| nrfA | 9.008334295499482 |
| degU | 11.323834116598489 |
| rBAM | 22.89917356394677 |
| Landy | 6.622807017543858 |Figure S10. IAA production of FZB42 and nrfA, degU, and RBAM_17410 (rBAM) mutant strains in absence (-T) and presence (+T) of Trp. Landy: medium control.
